# LncRNA MIR4435-2HG drives cancer progression by modulating cell cycle regulators and mTOR signaling in stroma-enriched subtypes of urothelial carcinoma of the bladder

**DOI:** 10.1007/s13402-023-00826-5

**Published:** 2023-06-24

**Authors:** Lu Pei, Dong Yan, Qingqing He, Jianqiu Kong, Meihua Yang, Honglian Ruan, Qiongqiong Lin, Lifang Huang, Jian Huang, Tianxin Lin, Haide Qin

**Affiliations:** 1 Guangdong Provincial Key Laboratory of Malignant Tumor Epigenetics and Gene Regulation, Sun Yat-Sen Memorial Hospital, Sun Yat-Sen University, Guangzhou, China; 2 Department of Urology, Sun Yat-Sen Memorial Hospital, Sun Yat-Sen University, Guangzhou, China; 3 Department of Urology, The First Affiliated Hospital of Nanchang University, Nanchang, China; 4 School of Public Health, Guangzhou Medical University, Guangzhou, China; 5 Department of Pathology, The Second Affiliated Hospital & Yuying Children’s Hospital of Wenzhou Medical University, Wenzhou, China

**Keywords:** MIR4435-2HG, Urothelial carcinoma of the bladder (UCB), LncRNA, Molecular subtype, mTOR

## Abstract

**Background:**

The risk for recurrence and metastasis after treatment for urothelial carcinoma of the bladder (UCB) is high. Therefore, identifying efficient prognostic markers and novel therapeutic targets is urgently needed. Several long noncoding RNAs (lncRNAs) have been reported to be correlated with UCB progression. In this study, we found that the subtype-specific lncRNA MIR4435-2 host gene (MIR4435-2HG) plays a novel oncogenic role in UCB.

**Methods:**

RNA-Seq data of TCGA/BLCA were analyzed. The expression of MIR4435-2HG was measured by qRT-PCR in 16 pairs of bladder cancer tissues and adjacent normal tissues. The clinical relecance of MIR4435-2HG was validated via in situ hybridization performed on an in-house cohort of 116 UCB patient samples. RNA pull-down followed by mass spectrometry was performed to identify MIR4435-2HG-binding proteins. To identify signaling pathways involved in MIR4435-2HG activity, comprehensive *in vitro* and *in vivo* studies and RNA-Seq assays were performed using UCB cells in which MIR4435-2HG expression was knocked down or exogenously overexpressed. In addition, we performed RNA immunoprecipitation and Western blot analyses to validate the identified MIR4435-2HG-binding proteins and to determine the molecular mechanisms by which MIR4435-2HG promotes UCB progression.

**Results:**

We found that MIR4435-2HG was significantly upregulated in the stromal-enriched subtype of UCB. Increased MIR4435-2HG expression was positively correlated with a high histological grade, advanced T stages, larger tumors, lymph node metastasis and a poor prognosis. *In vitro* experiments revealed that MIR4435-2HG expression silencing suppressed cell proliferation and induced apoptosis. Inhibition of MIR4434-2HG delayed xenograft tumor growth, while MIR4435-2HG overexpression reversed the MIR4435-2HG silencing-induced inhibition of UCB tumor phenotype acquisition. Mechanistically, we found that MIR4435-2HG positively regulated the expression of a variety of cell cycle regulators, including BRCA2 and CCND1. Knocking down MIR4435-2HG increased the sensitivity of tumor cells to the VEGFR inhibitor cediranib. Furthermore, we found that MIR4435-2HG regulated mTOR signaling and epithelial-mesenchymal transition (EMT) signaling pathways by modulating the phosphorylation of mTOR, 70S6K and 4EBP1. Finally, we confirmed that MIR4435-2HG enhances tumor metastasis through regulation of the EMT pathway.

**Conclusions:**

Our data indicate that upregulated MIR4435-2HG expression levels are significantly correlated with a poor prognosis of UCB patients. MIR4435-2HG promotes bladder cancer progression, mediates cell cycle (de)regulation and modulates mTOR signaling. MIR4435-2HG is an oncogenic lncRNA in UCB that may serve as a diagnostic and therapeutic target.

**Supplementary Information:**

The online version contains supplementary material available at 10.1007/s13402-023-00826-5.

## Introduction

Urothelial carcinoma of the bladder (UCB) is one of the most common urological malignancies with a high incidence and mortality worldwide [1, 2]. The prognosis of UCB patients remains poor due to high recurrence metastasis rates [3, 4]. At present, surgical treatment, chemotherapy and immunotherapy are the main treatments for UCB, but their efficacy is limited [5]. Therefore, identifying efficient prognostic markers and therapeutic targets for developing novel approaches for the treatment of UCB is important.

UCB is characterized by marked heterogeneity. Two to seven molecular UCB subtypes have been reported. The Cancer Genome Atlas (TCGA) presents five subtypes of UCB defined on the basis of distinct clinical features: the luminal-papillary, luminal, basal-squamous, luminal-infiltrated and neuronal subtypes. The invasive subtypes are strongly correlated with an enrichment of stromal cells and lymphocyte infiltration into tumor components [6]. Moreover, a low tumor-mutation burden and stromal enrichment have been reported to be indicators correlated with resistance to anti-PD-L1 antibody-based immune therapy [7]. Elucidating the molecular mechanisms underlying the development of UCB subtypes and the mechanistic interactions among cells in the tumor microenvironment (TME) is significant for identifying specific biomarkers and therapeutic targets for personalized medicine.

Long noncoding RNAs (lncRNAs) are specifically expressed based on the basis of the biological state of a tissue and are recognized as important regulatory factors because they can participate in multiple biological processes, including metastasis, autophagy, oxidative stress and immune suppression in cancer [8]. Several studies have suggested that lncRNAs, such as H19, DLX6-AS1 and BLACAT are closely related to the development of UCB, and that their abnormal expression may affect tumor growth, recurrence and metastasis [9, 11]. LncRNAs can be used as biomarkers and/or therapeutic targets for cancer [6, 12]. Importantly, lncRNAs have been shown to be concordant with mRNA-based subtypes in bladder cancer [6]. However, to date, the functional lncRNAs that drive subtype-specific cancer progression remain poorly understood.

In this study, we identified a unique lncRNA, the MIR4435-2 host gene (MIR4435-2HG), which was consistently upregulated in UCB tumors and correlated with a poor prognosis of UCB patients. A recent study on MIR4435-2HG in bladder cancer suggested that MIR4435-2HG may be correlated with the prognosis of UCB and its treatment response to BCG using bio-informatics [13]. An additional concurrent study explored the biological functions of MIR4435-2HG in bladder cancer cell lines [14]. As yet, however, the molecular mechanisms of MIR4435-2HG in promoting UCB remain not fully understood, especially the rolethat its elevated expression may play in specific subtypes of UCB. More specifically, it is unclear whether or not MIR4435-2HG can directly bind to its protein targets to exert its functions and whether it may play as a transcriptional (co-)activator to regulate its downstream target genes. Therefore, clinical analysis of large UCB samples and in-depth molecular mechanistic studies are warranted to underscore a role of MIR4435-2HG as a diagnostic marker and potential therapeutic target.

Interestingly, we found that MIR4435-2HG was specifically upregulated in the stroma-enriched subtype of UCB, including basal/squamous and luminal-infiltrated subtype, which is characterized by a substantial enrichment of stromal cells and immune cells in the TME. We further characterized the biological functions of the lncRNA *in vitro* in cell lines and in a nude mouse model. Our data indicate that MIR4435-2HG may govern the regulatory axes of the cell cycle, modulate the mechanistic target of rapamycin kinase (mTOR) signaling pathway, and regulate the treatment response of UCB to rapamycin. MIR4435-2HG may serve as a potential therapeutic target in UCB.

## Methods

### Study subjects and clinical data collection

A total of 116 formalin-fixed paraffin-embedded (FFPE) UCB tissue samples were obtained from Sun Yat-sen Memorial Hospital, Sun Yat-sen University (Guangzhou, China). In addition, a total of 16 UCB fresh tissue samples and paired adjacent tissue samples were obtained from patients who had undergone surgery at Sun Yat-sen Memorial Hospital, Sun Yat-sen University (Guangzhou, China). Detailed clinical information of the study subjects is provided in Supplementary Table S1. All human tissue samples were taken after a written informed consent was obtained from all patients, and this study was approved by the Research Ethics Committee of Sun Yat-sen Memorial Hospital. The study protocol was approved by the Research Ethics Committee of Sun Yat-sen Memorial Hospital.

### *In situ* hybridization (ISH)

A total of 116 FFPE tissue samples were sliced in preparation for ISH using MIR4435-2HG as a probe (Genebio, Shanghai) according to the manufacturer’s instructions. A detailed protocol of the ISH can be found in the Supplementary Information file. The sections were observed under a microscope (Nikon, Japan), and five specific regions were selected and captured. The staining intensity of MIR4435-2HG was scored using the H-score method, and the expression scores were calculated by a pathologist using standard methods.

### Cell culture

The cell lines used in this study included the UM-UC-3 and T24 human UCB celllines and the human embryonic kidney cell line HEK-293T, purchased from the American Type Culture Collection (ATCC, Manassas, VA, USA). The T24 cells were cultured in RPMI-1640 medium (GIBCO, Gaithersburg, MD, USA), and the UM-UC-3 and HEK-293T cells were cultured in DMEM medium (GIBCO, Gaithersburg, MD, USA). Both media were supplemented with 10% fetal bovine serum (FBS; Biological Industries, Beit Haemek, Israel) and antibiotics (100 U/ml penicillin and 100 mg/ml streptomycin) (Gibco, Gaithersburg, MD, USA), and the cells were maintained in a humidified atmosphere with 5% CO_2_ at 37 °C.

### RNA isolation and quantitative reverse transcription PCR (qRT-PCR)

Total RNA was extracted from tissues and UCB cells using RNAiso Plus (Takara Bio, Shiga, Japan). RNAs from nuclear and cytoplasmic extracts was isolated using a nuclear and cytoplasmic extraction kit (Life Technologies, Camarillo, CA, USA) according to the manufacturer’s instructions. One microgram of purified total RNA was reverse transcribed into cDNA using random primers with the reverse transcription kit PrimeScript RT Master Mix (TaKaRa Bio, Shiga, Japan). qRT-PCR analyses were performed using TB Green Premix Ex TaqII (Takara Bio, Shiga, Japan) on a Quantstudio Dx system (Applied Biosystems, Singapore). The thermocycling conditions were 95 °C for 5 min to preheat, followed by 40 cycles of 95 °C for 10 s and 60 °C for 30 s to amplify the signals. Relative quantification was carried out using the comparative cycle threshold (CT) (2^−ΔΔCT^) method. Gene expression was normalized to the expression of glyceraldehyde-3-phosphate dehydrogenase (GAPDH). The specific primer sequences used are listed in Supplementary Table S2.

### RNA interference

T24 or UM-UC-3 cells were seeded in 6-well plates and transfected with small interfering RNAs (siRNAs) using Lipofectamine RNAiMax (Life Technologies, Waltham, MA, USA) for 24 h. siRNAs targeting MIR4435-2HG and negative control siRNAs were purchased from GenePharma (Shanghai, China). Cells were harvested 48 h and 72 h after transfection and subjected to qRT-PCR and western blot analysis, respectively. The sequences of the siRNAs were as follows: silencing control (siCtrl), UUCUCCGAACGUGUCACGUTT; si-MIR4435-2HG#1, CCAGAACACCCACAAGCUUTT; and si-MIR4435-2HG#2, GGACUUGACUUAAUGCCUUTT.

### Plasmid construction and transfection

To establish stable knockdown cell lines, short hairpin RNA (shRNA) sequences specifically targeting MIR4435-2HG were cloned into GV248-Puro (GeneChem, Shanghai, China). ShRNA plasmid, along with psPAX2 and PMD2.G plasmids, were transfected into HEK-293 T cells to package lentivirus. To obtain an overexpression plasmid, the MIR4435-2HG sequence was cloned into a pcDNA3.1( +) vector (IGE Biotechnology, Guangzhou, China). The plasmid vectors were transfected into UCB cells using X-tremeGENE HP DNA transfection reagent (Sigma, St. Louis, MO, USA). The transfection procedure was performed according to the manufacturer’s protocol. The cells were harvested 48 h and 72 h after transfection and subjected to qRT-PCR and Western blot analysis, respectively.

### Cell viability assay

T24 and UM-UC-3 cells transfected with si-MIR4435-2HGs or pcDNA3.1( +)-MIR4435-2HG were seeded in 96-well plates at a density of 1 × 10^3^ cells per well and cultured for 6 days. Next, 10 microliters of ﻿cell counting kit-8 (CCK-8) reagent (Dojindo Laboratories, Japan) was added to each well, and the absorbance was read 2 h later and at 24 h intervals at 450 nm on a Spark 10 M microplate reader (Tecan, Austria).

### Colony formation assay

In total 1 × 10^3^ siRNA- or plasmid- transfected cells were cultured in 6-well plates and maintained in the corresponding complete medium, which was replaced every 3 days. After 2 weeks, the colonies were fixed with 4% paraformaldehyde (Servicebio, Wuhan, China) for 20 min and stained with 0.1% crystal violet (Sigma-Aldrich, Darmstadt, Germany) for 15 min. Colonies were counted and imaged using a vSpot Spectrum device (AID, Germany).

### Cell migration and invasion assays

Cell migration and invasion were evaluated by performing Transwell assays. Transwell chambers with a pore size of 8.0 μm (Millipore, Darmstadt, Germany) were used for these assays. UM-UC-3 cells (8 × 10^4^) and T24 cells (6 × 10^4^) were evenly suspended in serum-free medium and placed in the upper Transwell chambers with or without Matrigel matrix (Corning, USA) in 24-well plates. Medium containing 10% FBS was added to the lower chambers as a chemoattractant. After 20 h and 8 h culture of the UM-UC-3 and T24 cells, respectively, the Transwell chambers were removed from the plates, and the cells were fixed with 4% paraformaldehyde (Servicebio) for 20 min and stained with 0.1% crystal violet (Sigma-Aldrich) for 15 min. Then, the migrated cells were evaluated under an orthographic-view microscope (Nikon, Japan) and cells in five randomly selected fields of view were counted.

### Flow cytometry 

T24 and UM-UC-3 cells transfected with si-MIR4435-2HGs were harvested 48 h after transfection by trypsinization and then centrifuged. After double staining with FITC-Annexin V and propidium iodide (PI) in the dark for 20 min using a fluorescein isothiocyanate isomer (FITC) Annexin V Apoptosis Detection Kit (Key GENE Bio, Nanjing, China), the cells were analyzed using a CytoFLEX apparatus (Beckman, USA) equipped with CytExpert software. The relative numbers of early apoptotic cells were compared between the siMIR4435-2HG-transfected and negative control cell populations. Cells used for cell cycle analysis were washed twice with ice-cold PBS and fixed in 75% ice-cold ethanol at 4 °C overnight. The fixed cells were then treated with RNaseA in a water bath at 37 °C for 30 min and stained with PI using a cell cycle assay kit (Elabscience, Wuhan, China). The percentages of cells in G0/G1 phase, S phase, and G2/M phase were counted and compared using CytoFLEX.

### Nude mouse xenograft models

The animal experimental procedures in our study were approved by the Animal Ethics Committee of Sun Yat-sen University. Four-week-old male nude BALB/c mice, purchased from Beijing Vital River, were maintained under specific pathogen-free (SPF) conditions. To evaluate the role of MIR4435-2HG in the tumorigenesis of UCB, a subcutaneous neoplasia model was applied. To this end, stably MIR4435-2HG expressing T24 cells were constructed by lentivirus transduction as described above. The quarantined nude mice were randomly divided equally into two groups (*n* = 10 per group). A total of 5 × 10^6^ constructed T24 cells were suspended in 100 μl PBS and injected subcutaneously into the right dorsal area of the mice. Tumor sizes were measured every 3 days and the volumes were calculated using the formula: length × width^2^ × 0.5. Twenty-eight days post-injection, the mice were euthanized, and the tumors were dissected and fixed in formalin for further analysis.

### RNA-Seq

T24 and UM-UC-3 cells were transfected with si-MIR4435-2HGs or control siRNA for 48 h. Next, total RNA was then extracted from the cells using TRIzol reagent and subjected to RNA-Seq, according to the manufacturer’s instructions. Library construction and RNA-sequencing were conducted by RiboBio (Guangzhou, China). The libraries were sequenced on an Illumina HiSeq 3000 platform, and 150-bp paired-end reads were generated. ENSEMBL data (http://www.ensembl.org/index.html) were downloaded and used as reference genomes. HISAT2 was used to align clean reads to the reference genome. edgeR was used to analyze differential gene expression between the control and si-MIR4435-2HGs groups.

### Subcellular fractionation

To separate nuclear and cytoplasmic fractions from T24 and UM-UC-3 cells, a PARIS Kit (Life Technologies, Carlsbad, CA) was used according to the manufacturer’s instructions. Briefly, 10^7^ UCB cells were harvested by trypsinization, centrifuged and lysed using 600 μl of cell lysis buffer. The cells were then centrifuged at 3,000 × g for 15 min, and the supernatant containing the cytoplasmic components was carefully aspirated from the nuclear pellet, which was retained for subsequent RNA extraction.

### RNA pull-down assay and mass spectrometry

A pcDNA3.1( +)-MIR4435-2HG vector was cleaved by the restriction enzyme EcoRI (New England Biolabs, USA) at 37 °C for 15 min. Next, the digestion products were purified using a QIAquick PCR Purification Kit (Qiagen, Germany). MIR4435-2HG was transcribed from the vector using a TranscriptAid T7 High Yield Transcription Kit (Thermo Scientific, Waltham, MA, USA) and then purified *in vitro* using a GeneJET RNA purification kit (Thermo Scientific, Waltham, MA, USA). The 3’ end of MIR4435-2HG was biotin-labeled using a Pierce RNA 3’ End Desthiobiotinylation Kit (Thermo Scientific, Waltham, MA, USA) according to the manufacturer’s instructions. For the RNA pulldown assay, 50 pmol biotinylated RNA and 50 µl RNA capture buffer were incubated with magnetic beads for 30 min. T24 cell lysates were then incubated with Master Mix of RNA–Protein Binding Reaction and magnetic beads at 4 °C for 1 h. RNA–protein complexes were isolated from the magnetic beads using Elution Buffer and boiled in loading buffer at 100 °C for 10 min. The retrieved proteins were identified by mass spectrometry at the Bioinformatics and Omics Center, Sun Yat-Sen Memorial Hospital, Sun Yat-Sen University.

### RNA immunoprecipitation (RIP) assay

T24 and UM-UC-3 cells were scraped off the culture plates when the cell density reached 80–90% confluency and then lysed in complete RIP lysis buffer using a Magna RIP kit (Millipore, Billerica, MA). T24 cell extracts were incubated with RIP immunoprecipitation buffer containing magnetic beads conjugated with antibodies or control IgG overnight at 4 °C on a rotary mixer. The beads were washed with wash buffer 8 times, after which the complexes were incubated with Proteinase K with rotation at 800 rpm and 55 °C for 30 min to remove proteins. The preserved RNA was then extracted using TRIzol reagent and purified. The RNA concentration was measured using a NanoDrop spectrophotometer (Thermo Scientific, Waltham, MA, USA) and then analyzed via qRT‒PCR. GAPDH was used as a nonspecific control, and IgG was used as the negative control.

### Hemoxylin and eosin (H&E) staining and immunohistochemistry (IHC) 

The staining cells for these analyses was performed as previously described [15]. Anti-Ki67 (1:500, Zhongshan Bio-Tech, Beijing, China) and anti-BRCA2 (1:100, Cell Signaling Technology) antibodies were used for these assays.

### Western blot assay

Cells were lysed completely in ice-cold strong radio immunoprecipitation assay (RIPA) lysis buffer (Beyotime, Shanghai, China). Protein samples (20 μg) were electrophoresed through 10% polyacrylamide gels (Epizyme, Shanghai, China) at a constant voltage and then transferred to polyvinylidene difluoride (PVDF) membranes (Merck Millipore, Darmstadt, Germany) under constant current. The membranes were blocked with 5% nonfat milk in tris buffered saline with tween-20 (TBST) for 1 h and then incubated with diluted primary antibodies at 4 °C overnight. The next day, the membranes were washed 4 times with TBST and then incubated with diluted goat anti-rabbit IgG-HRP secondary antibody (CWBio, Beijing, China) for 1 h. Specific bands were cut from the membranes, identified on the basis of the markers, and detected by enhanced chemiluminescence (Thermo Fisher Scientific, USA) using SmartChemi 910 plus (Sinsitech, Beijing, China). The antibodies used in this study are listed in Supplementary Table S3.

### Bioinformatics analysis and statistical analyses

Gene expression data were obtained from the TCGA database (https://tcga-data.nci.nih.gov/tcga/). Stromal score and Immune score were calculated from the expression data using the ESTIMATE webtool (https://bioinformatics.mdanderson.org/estimate/). To generate a Heatmap visualization for the array data, the webtool accessible at http://www1.heatmapper.ca/expression/ was utilized. To perform functional enrichment analysis, we employed the WebGestalt tool (https://www.webgestalt.org/). Network analysis was conducted using STRING (https://cn.string-db.org/). We used in-house R scripts for data analysis and visualization. Model construction, survival analysis, and correlation analysis were performed using in-house scripts based on R packages, including glmnet, survival, survminer, survivalROC and ggpubr. Chi-square analysis was used to determine the degree of association of MIR4435-2HG expression levels with clinical variables.

For determining the significance of differences in the *in vitro* and *in vivo* experimental data, Student’s *t* test (two-tailed), one-way analysis of variance and the Mann‒Whitney *U* test were performed. *P* values less than 0.05 were considered to be significant. We used GraphPad Prism version 8 software for generic visualization.

## Results

### MIR4435-2HG expression is upregulated in UCB cells

**Fig. 1:**
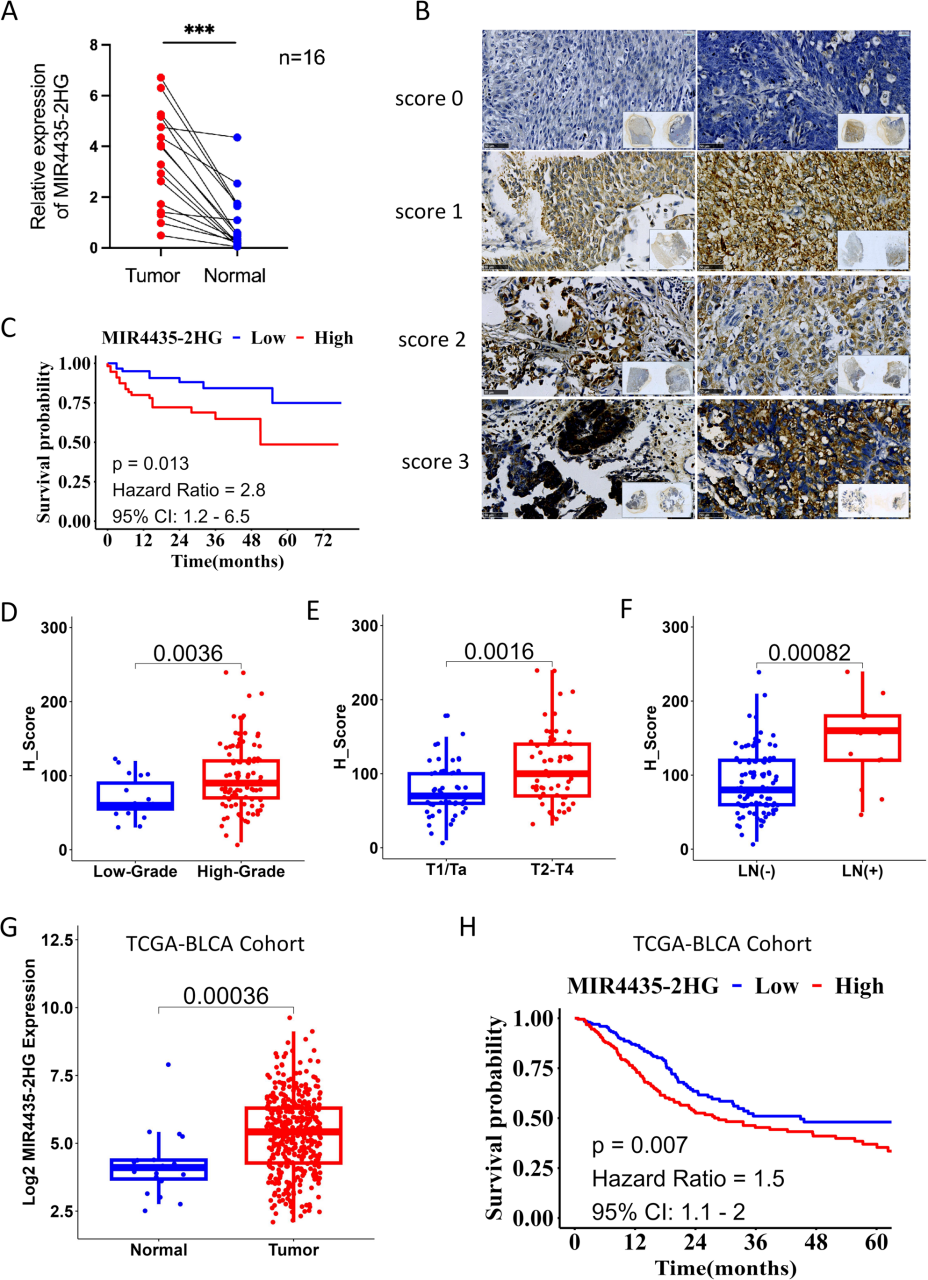
Relative MIR4435-2HG expression in UCB tissues and its clinical significance. **A** MIR4435-2HG expression in bladder cancer tissues (*n* = 16) compared with normal tissues (*n* = 16) examined by qRT-PCR and normalized to GAPDH expression. **B** Expression of MIR4435-2HG in 116 UCB tissues detected by in situ hybridization. Score from 0 to 3. **C** Kaplan–Meier overall survival curves according to MIR4435-2HG expression levels. **D** H-scores of MIR4435-2HG in patients with different tumor grades. **E** H-scores of MIR4435-2HG in patients with different clinical T stages. **F** H-scores of MIR4435-2HG in patients with different LN statues. **G** Expression of MIR4435-2HG in the TCGA/BLCA cohort. **H** Kaplan–Meier overall survival curves according to MIR4435-2HG expression levels in the TCGA/BLCA cohort.*p < 0.05, ***p< 0.001

Through an analysis of the TCGA/BLCA lncRNA data, we found that the expression levels of MIR4435-2HG were significantly upregulated in UCB tumor tissues compared with those in normal tissues. To validate these results, we performed qRT-PCR to analyze the expression of MIR4435-2HG in UCB tissues and adjacent normal tissues taken from 16 patients. We confirmed that MIR4435-2HG was upregulated in UCB tissues compared to normal adjacent tissues (Fig. 1A). Subsequent ISH revealed that MIR4435-2HG was distributed mainly in the cytoplasm in the UCB cells (Fig. 1B).

Next, to assess correlations between MIR4435-2HG expression and patient clinicopathological characteristics, we performed an clinical analysis of our cohort of patients (*n* = 116). We found that higher expression levels of MIR4435-2HG were significantly associated with a high histological grade (*p* = 0.043), an advanced T stage (*p* = 0.014) and positive lymph node metastasis (*p* = 0.039) (Table 1). A Kaplan–Meier survival analysis was subsequently performed to examine the association between MIR4435-2HG expression levels and UCB prognosis. We found that patients with higher MIR4435-2HG expression levels exhibited significantly shorter overall survival times than those with lower expression levels (*p* = 0.013) (Fig. 1C). The expression analysis also indicated that high tumor grade, advanced T stage (T2-T4) and positive LN metastasis were associated with high MIR4435-2HG expression (Fig. 1D–F).

**Table 1:** Association analysis of MIR4435-2HG expression levels with clinical-pathological variables of urothelial carcinoma patients

Variables	MIR4435-2HG(n, %)			OR(95%CI)	*p*-value
	Low expression	High expression	Total		
Gender					
Female	9 (15.0)	6 (10.7)	15 (12.9)		
Male	51 (85.0)	50 (89.3)	101 (87.1)	1.47(0.49–4.67)	0.494
Age (years)					
≤ 65	31 (51.7)	30 (53.6)	61 (52.6)		
> 65	29 (48.3)	26 (46.4)	55 (47.4)	0.93(0.45–1.92)	0.837
Stage					
T1/Ta	33 (55.0)	18 (32.1)	51 (44.0)		
T2-T4	27 (45.0)	38 (67.9)	65 (56.0)	2.58(1.22–5.58)	0.014
Nodal_status					
0	57 (95.0)	46 (82.1)	103 (88.8)		
1	3 (5.0)	10 (17.9)	13 (11.2)	4.13(1.18–19.20)	0.039
Grade					
Low	14 (23.3)	5 (8.9)	19 (16.4)		
High	46 (76.7)	51 (91.1)	97 (83.6)	3.10(1.09–10.20)	0.043

A survival analysis based on TCGA data revealed that UCB patients with a high MIR4435-2HG expression exhibited a shorter overall survival rate than those with low expression of MIR4435-2HG (Fig. 1G and H). Therefore, we conclude that MIR4435-2HG may contribute to the aggressiveness of tumors, a finding that warrants further study.

### MIR4435-2HG expression is elevated in aggressive molecular subtypes of UCB

**Fig. 2:**
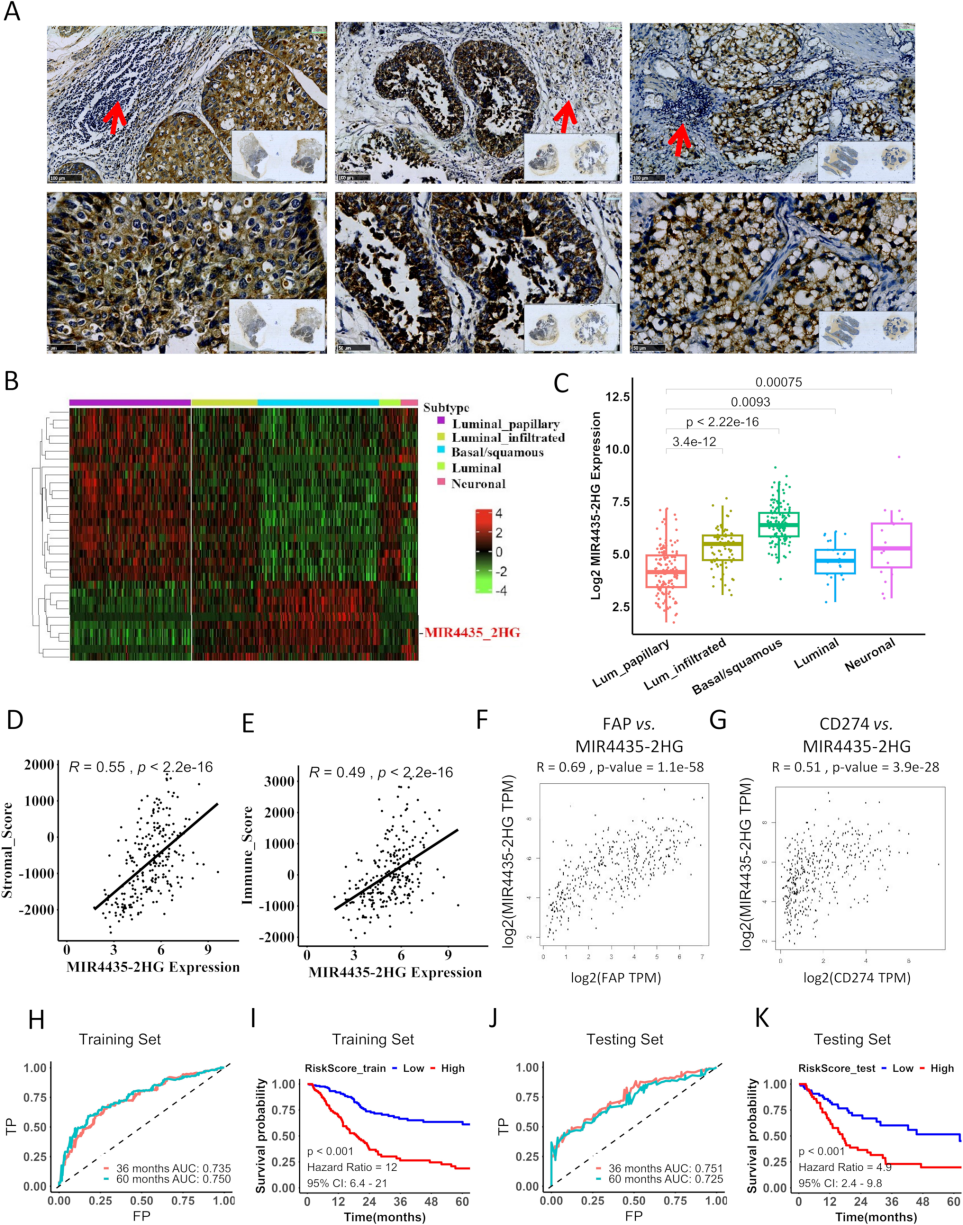
Expression of MIR4435-2HG correlates with the stroma-enriched subtype of UCB. **A** Immune cell infiltration (red arrows) in stroma-enriched tumors with a high MIR4435-2HG expression. **B-C** The expression of MIR4435-2HG is significantly elevated in the basal/squamous subtype and luminal-infiltrated subtype of UCB. **D-E** Scatter plots to illustrate the correlation of the expression of MIR4435-2HG with the stromal score and the immune score of UCB. **F-G** Scatter plots to illustrate the correlation of MIR4435-2HG expression with stroma markers and immune cell markers. **H–K** Co-expression network of MIR4435-2HG predicting the survival of the UCB. Note, H indicates the ROC curves for the prediction of the survival in the training set; I indicates the HRs for the high- and low-risk groups in the training dataset; J indicates the ROC curves for the prediction of the survival in the test dataset; K indicates the HRs for the high- and low- risk groups in the test dataset

To obtain further insight into the involvement of MIR4435-2HG in UCB, we analyzed the expression of MIR4435-2HG in five molecular UCB subtype samples in the TCGA/BLCA dataset, i.e., the luminal-papillary, luminal, basal-squamous, luminal-infiltrated and neuronal subtypes. We found that MIR4435-2HG expression was higher in the basal-squamous subtype and the luminal-infiltrated subtype than in the luminal-papillary subtype (all *p* < 0.0001; Fig. 2A–2C). Due to a strong correlation of stromal enrichment and lymphocyte infiltration in the TME, the expression levels of MIR4435-2HG were also found to be significantly correlated with stromal/immune scores (Fig. 2D and E). Using gene expression profiling interactive analysis (GEPIA) we found that the expression of MIR4435-2HG and the stroma markers and immune cell markers was positively correlated (Fig. 2F and G; Supplementary Fig. S1).

A subsequent co-expression analysis revealed that the expression level of MIR4435-2HG was significantly correlated with that of epithelial-mesenchymal transition (EMT)-associated genes (Supplementary Fig. S2). Moreover, we found that the genes in the co-expression network efficiently predicted the survival of UCB patients (Fig. 2H–K; Supplementary Fig. S3). These results indicate that the expression of MIR4435-2HG is specifically correlates with stroma-enriched subtypes and immune cell infiltration in UCB.

### MIR4435-2HG silencing inhibits bladder cancer cell proliferation

**Fig. 3:**
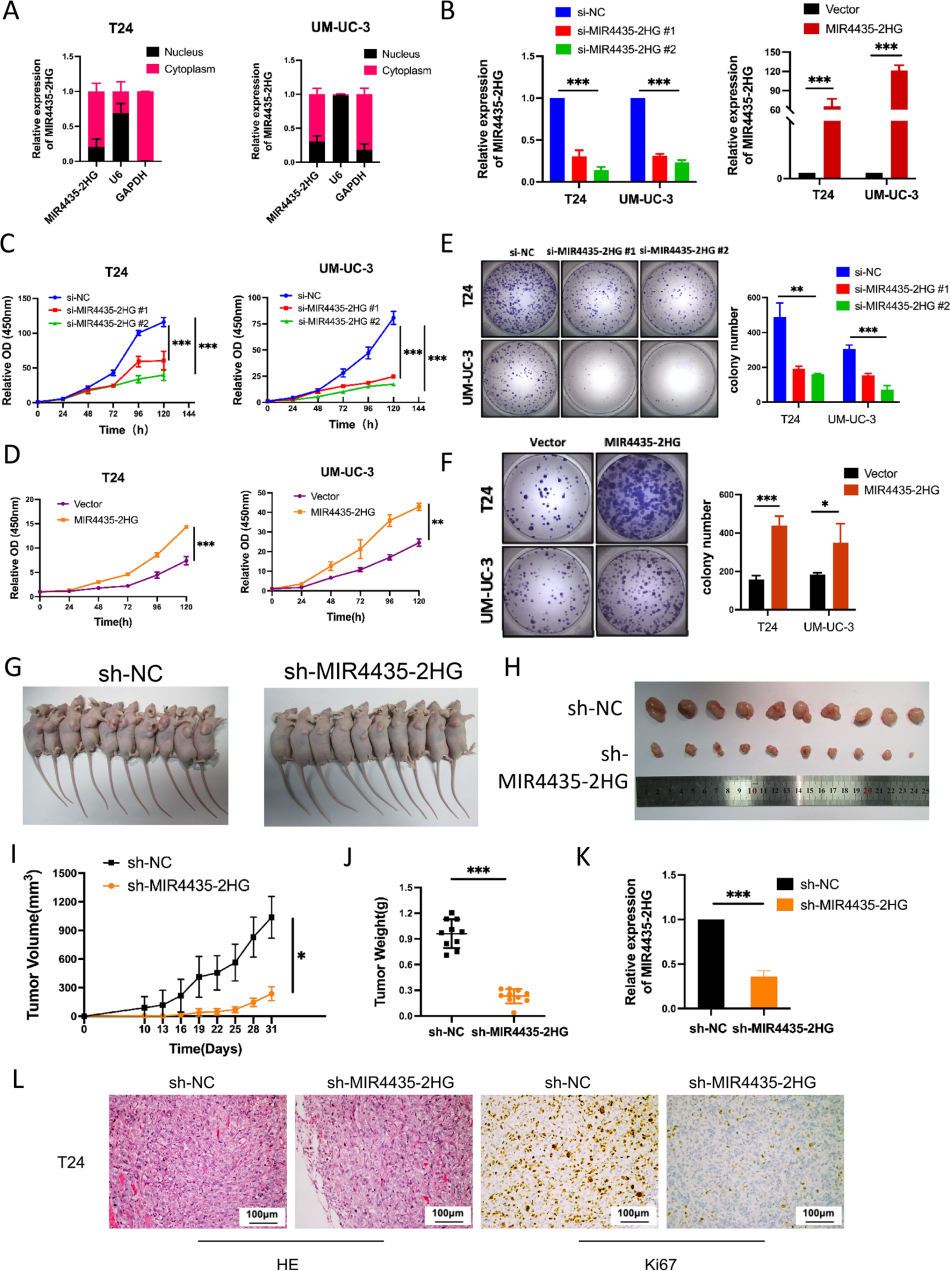
Effects of MIR4435-2HG on UCB cell proliferation *in vitro* and *in* *vivo*. **A** Nuclear fractionation experiment and qRT-PCR detection of the abundance of MIR4435-2HG in the nucleus and the cytoplasm. GAPDH was used as positive control for the cytoplasm, and U6 was positive control for the nucleus. **B** Downregulation and upregulation of MIR4435-2HG in T24 and UM-UC-3 cells after siRNA or pcDNA3.1( +)-MIR4435-2HG plasmid transfection, respectively. **C-D** CCK-8 assay-based detection of the viability of si-MIR4435-2HG or pcDNA-MIR4435-2HG transfected T24 and UM-UC-3 cells. **E–F** Colony formation assay-based determination of the proliferation of transfected UCB cells. Colonies were counted and captured. Values are presented as the mean ± s.d of three independent experiments. **G-H** T24 cells transfected with empty vector or sh-MIR4435-2HG were injected into the nude mice (*n* = 10). Tumors before and after inoculation of the nude mice. **I** Tumor volumes were calculated every 3 days. **J** Tumor weights were measured after tumor removal. **K** qRT-PCR was used to examine the average expression of MIR4435-2HG in tumor tissues formed from T24/empty vector and T24/sh-MIR4435-2HG cells. **L** Ki67 was detected by immunohistochemistry. *p < 0.05, **P < 0.01, ***p < 0.001

To explore the potential role played by MIR4435-2HG in UCB cells, we assessed the distribution of MIR4435-2HG in T24 and UM-UC-3 cells by subcellular fractionation and qRT-PCR. We found that MIR4435-2HG was distributed mainly in the cytoplasm in the UCB cells (Fig. 3A). Next, we synthesized two siRNAs to silence the expression of MIR4435-2HG and used qRT-PCR to confirm that MIR4435-2HG was markedly downregulated in UM-UC-3 and T24 cells after transfection. We also transfected T24 and UM-UC-3 cells with the pcDNA3.1( +)-MIR4435-2HG vector to exogenously overexpress MIR4435-2HG. The expression of MIR4435-2HG was found to be markedly upregulated in cells carrying pcDNA3.1( +)-MIR4435-2HG compared with those carrying the empty vector (Fig. 3B).

We next examined the role played by MIR4435-2HG in the development of UCB. Using CCK-8 assays,we found that the viability of T24 and UM-UC-3 cells was significantly decreased after MIR4435-2HG expression down-regulation (Fig. 3C). In contrast, we found that T24 and UM-UC-3 cells transfected with pcDNA3.1( +)-MIR4435-2HG exhibited a higher viability than the negative controls (Fig. 3D). Additionally, the colony formation ability of T24 and UM-UC-3 cells with MIR4435-2HG knocked down was found to be profoundly attenuated (Fig. 3E), while the colony forming capacity was markedly increased in MIR4435-2HG-overexpressing T24 and UM-UC-3 cells (Fig. 3F). These findings indicate that MIR4435-2HG may play an oncogenic role in UCB.

### MIR4435-2HG promotes the tumorigenesis of bladder cancer cells *in vivo*

Next, we constructed a xenograft mouse model to verify the oncogenic role played by MIR4435-2HG in UCB. After stable transfection with sh-MIR4435-2HG or empty vector, T24 cells were injected into nude mice. We found that MIR4435-2HG knockdown substantially decreased tumor growth in nude mice. The tumors that formed in the sh-MIR4435-2HG group were smaller than those in the empty vector group (Fig. 3G, H). In addition, the tumor volumes and weights in the sh-MIR4435-2HG group were less than those in the empty vector group (Fig. 3I and J). Concordantly, we found using qRT-PCR that the expression of MIR4435-2HG in tumor tissues obtained from the sh-MIR4435-2HG group was lower than that obtained from the empty vector group (Fig. 3K). The proportion of tumor-free mice in the sh-MIR4435-2HG group was higher than that in the sh-NC treatment group. Moreover, we found that MIR4435-2HG knockdown inhibited Ki67 expression *in vivo* (Fig. 3L). Collectively, these data indicate that MIR4435-2HG is involved in UCB oncogenesis.

### MIR4435-2HG regulates cell cycle progression in UCB cells

**Fig. 4:**
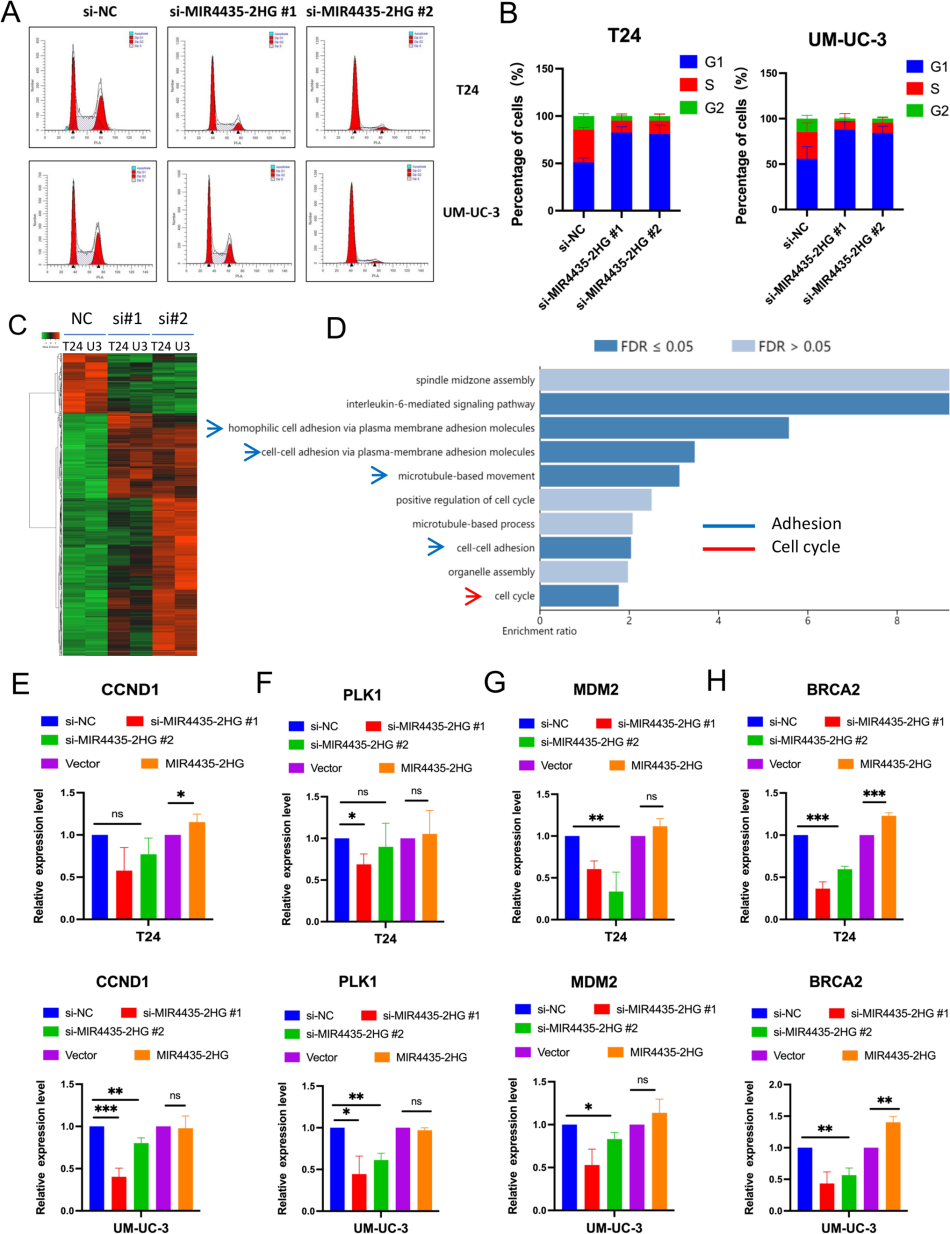
MIR4435-2HG affectes cell cycle progression in UCB cells. T24 and UM-UC-3 cells were transfected with si-NC or si-MIR4435-2HG 1#, 2#. **A-B** Flow cytometry was performed to determine the cell cycle in T24 and UM-UC-3 cells. **C** A heatmap representing mRNA expression levels in the T24 and UM-UC-3 cells transfected with control or MIR4435-2HG siRNA for 48 h (U3: UM-UC-3). **D** Pathways for enrichment analysis of differential genes. **E–H** The differentially expressed genes detected by RNA-seq were verified in T24 and UM-UC-3 cells by qRT-PCR. *p < 0.05, **p < 0.01, ***p < 0.001

To explore whether MIR4435-2HG contributes to the regulation of the cell cycle in UCB cells, we performed a flow cytometry analysis to examine cell cycle progression in MIR4435-2HG-knockdown cells and compared the results with those in control cells. We found that the percentage of T24 and UM-UC-3 cells transfected with si-MIR4435-2HGs in the G1/G0 phase was higher than that of the control cells (Fig. 4A and B). These results indicate that MIR4435-2HG knockdown contributes to cell cycle arrest at the G1/S checkpoint.

To further explore the molecular mechanism underlying MIR4435-2HG-induced cell proliferation in UCB, RNA-Seq was performed to compare gene expression profiles between MIR4435-2HG-silenced T24 and UM-UC-3 cells and the respective control cells. We found that among the genes regulated by MIR4435-2HG (|fold change|> 2.0), many were associated with known cancer pathways (Fig. 4C), including multiple transcription factors (Supplementary Fig. S4). Notably, we found that *MDM2*, *BRCA2* and *PLK1* were significantly downregulated in MIR4435-2HG-silenced cells. An enrichment analysis subsequently revealed that the transcriptome alterations mediated by MIR4435-2HG were significantly enriched in cell cycle-related pathways (Fig. 4D). Moreover, using qRT-PCR, we confirmed that MIR4435-2HG overexpression enhanced the expression of multiple cell cycle master regulators, including *CCND1*, *MDM2* and *PLK1*. These results imply that MIR4435-2HG may regulate UCB cell proliferation by targeting several master regulators, including CCND1 (Fig. 4E–H).

Through RNA-Seq analysis of MIR4435-2HG knockdown cells, we found that *BRCA2* expression was most profoundly downregulated, as determined by fold-change in MIR4435-2HG-depleted cells, and that this gene was also one of the most highly upregulated ones in MIR4435-2HG -overexpressing T24 and UM-UC-3 cells. These data suggested that the transcription of BRCA2 may be regulated by MIR4435-2HG in UCB cells.

### MIR4435-2HG konckout induces UCB cell apoptosis and enhances the sensitivity of UCB cells to the VEGFR inhibitor cediranib

**Fig. 5:**
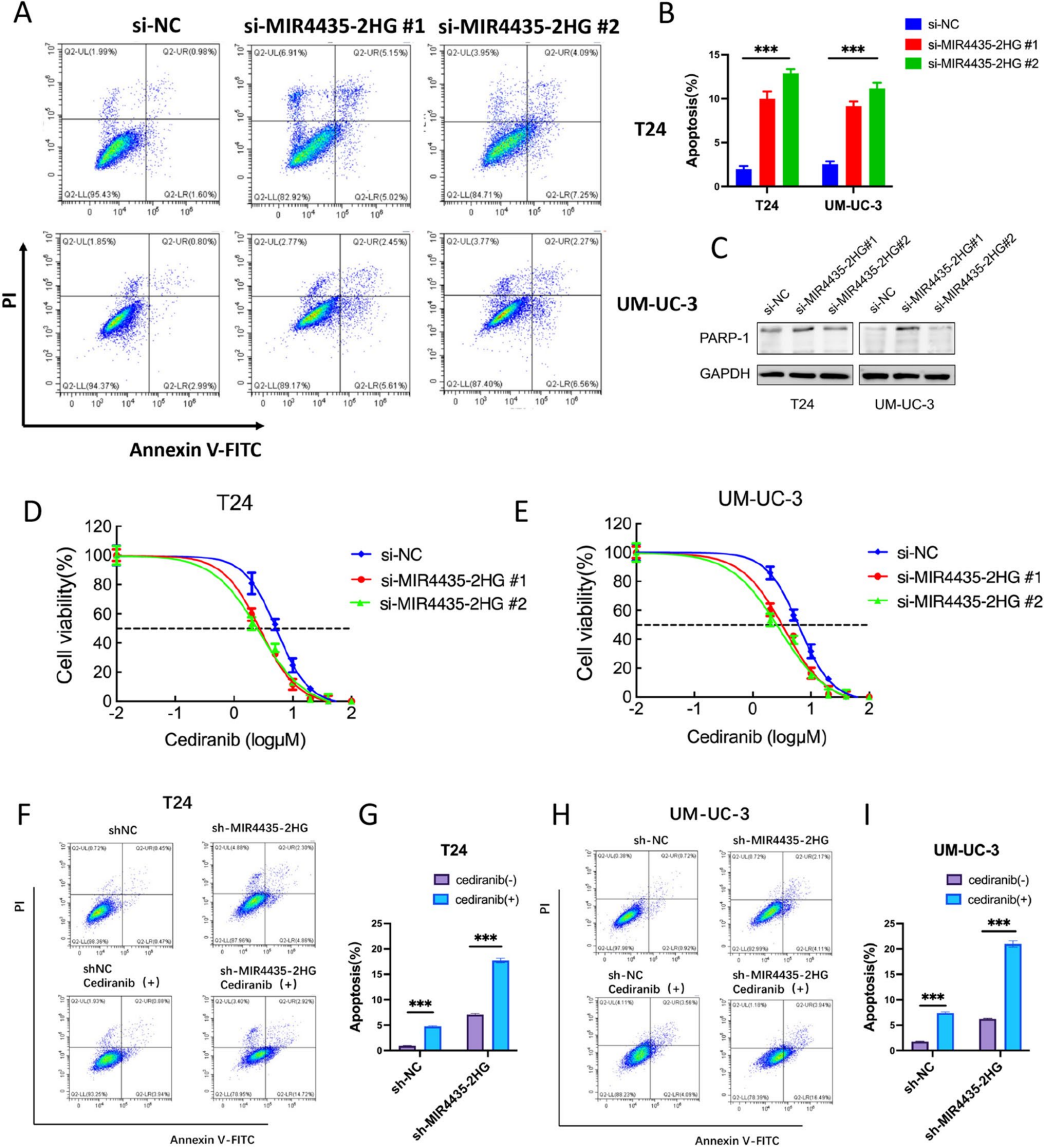
MIR4435-2HG affects the apoptosis in UCB cells. **A-B** Flow cytometry was performed to determine the apoptotic rates of cells. Values are presented as the mean ± s.d of three independent experiments. **C** Expression of PARP-1 detected by Western blotting in T24 and UM-UC-3 cells. **D-E** IC_50_ of cediranib in T24 and UM-UC-3 cells with or without si-MIR4435-2HG#1,2. **F-I** Flow cytometry was performed to detect the apoptotic rates of sh-MIR4435 T24 and UM-UC-3 cells with or without stimulation of cediranib. *p < 0.05, **p < 0.01, ***p < 0.001

To verify whether MIR4435-2HG can regulate cell apoptosis, we next set out to assess apoptotic fractions after introducing si-MIR4435-2HG into T24 and UM-UC-3 cells. We found that when si-MIR4435-2HG introduced, the proportion of cell apoptosis was significantly increased compared to controls (Fig. 5A–B). Concordantly, we found after Western blotting that the biomarker PARP-1 was significantly up-regulated (Fig. 5C). These data indicate that MIR4435-2HG knockdown enhances apoptosis in UCB cells.

Previous studies have shown that cediranib is a highly effective VEGFR (KDR) inhibitor and that cediranib targets BRCA2/RAD51 in tumor cells [16]. To investigate whether MIR4435-2HG regulates the effect of cediranib on BRCA2/RAD51, we compared *BRCA2* gene expression in MIR4435-2HG knockdown cells with that in control cells. We found that the IC_50_ of T24 and UM-UC-3 cells in the si-MIR4435-2HG group was significantly decreased, suggesting that knocking down MIR4435-2HG increased the sensitivity of the UCB cells to cediranib (Fig. 5D and E). A subsequent flow cytometry analysis revealed that the apoptotic rate of the cediranib( +)/sh-nc cells was higher than that of the cediranib(-)/sh-nc cells, and that the apoptotic rate of the cediranib( +)/sh-MIR4435-2HG cells was higher than that of the cediranib(-)/sh-MIR4435-2HG cells. A statistical analysis showed that the effect of cediranib on apoptosis significantly differed between the control group and the MIR4435-2HG-knockdown group (*p* < 0.001) (Fig. 5F–I). These results suggest that knocking down MIR4435-2HG increases the sensitivity of UCB cells to the inhibitor cediranib.

### MIR4435-2HG promotes the migration and invasion of bladder cancer cells by activating the EMT pathway

**Fig. 6:**
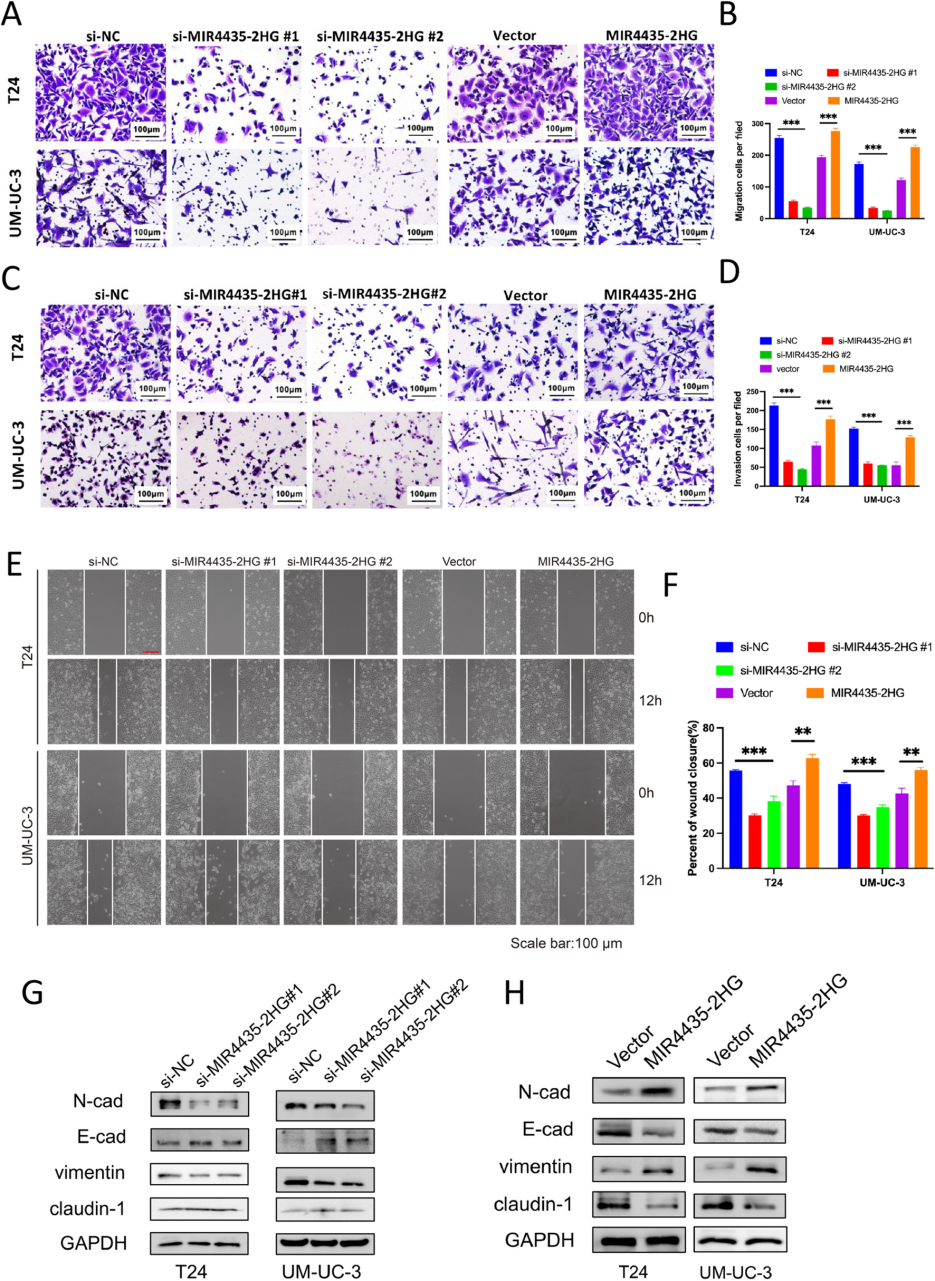
MIR4435-2HG promotes the migration and invasion of UCB cells through the EMT pathway. **A**, **B** Transwell assay showing changes in the migration ability of T24 and UM-UC-3 cells following knockdown or overexpression of MIR4435-2HG. **C**, **D** Transwell assay with Matrigel invasion chambers to evaluate changes in the invasion ability of T24 and UM-UC-3 cells following knockdown or overexpression of MIR4435-2HG. **E**, **F** Scratch wound healing assay showing cell migration after knockdown or overexpression of MIR4435-2HG in T24 and UM-UC-3 cells. **G** Western-blot analysis of the EMT signaling pathway in the MIR4435-2HG-depleted T24 and UM-UC-3 cells. **H** Western-blot analysis of the EMT signaling pathway in MIR4435-2HG overexpressing UCB cells

Using ISH, we found that in certain patients MIR4435-2HG was highly expressed in stromal cells and tumor cells. A subsequent co-expression analysis also showed that MIR4435-2HG expression was significantly correlated with stromal cell-related genes. Therefore, we speculated that elevated MIR4435-2HG levels in tumors may induce metastasis by regulating the EMT pathway. By performing scratch wound-healing and Transwell assays, we found that MIR4435-2HG silencing inhibited the migration, invasion and wound-healing capacity of UCB cells (Fig. 6A–F). In contrast, we found that MIR4435-2HG overexpression promoted the migration, invasion and wound-healing capacity of UCB cells. These results indicate that MIR4435-2HG promotes the migration and invasion of bladder cancer cells. Moreover, taking a Western blot approach, we analyzed the levels of EMT-related markers after MIR4435-2HG knockdown and found that the expression levels of E-cadherin and claudin1 were increased and that those of N-cadherin and vimentin were decreased after MIR4435-2HG knockdown (Fig. 6G), while MIR4435-2HG overexpression reversed the MIR4435-2HG silencing-induced acquisition (Fig. 6H). Taken together, these results suggest that MIR4435-2HG plays a key role in inducing the EMT in bladder cancer.

### MIR4435-2HG directly interacts with mTOR complex 1 and regulates the mTOR signaling pathway in UCB cells

**Fig. 7:**
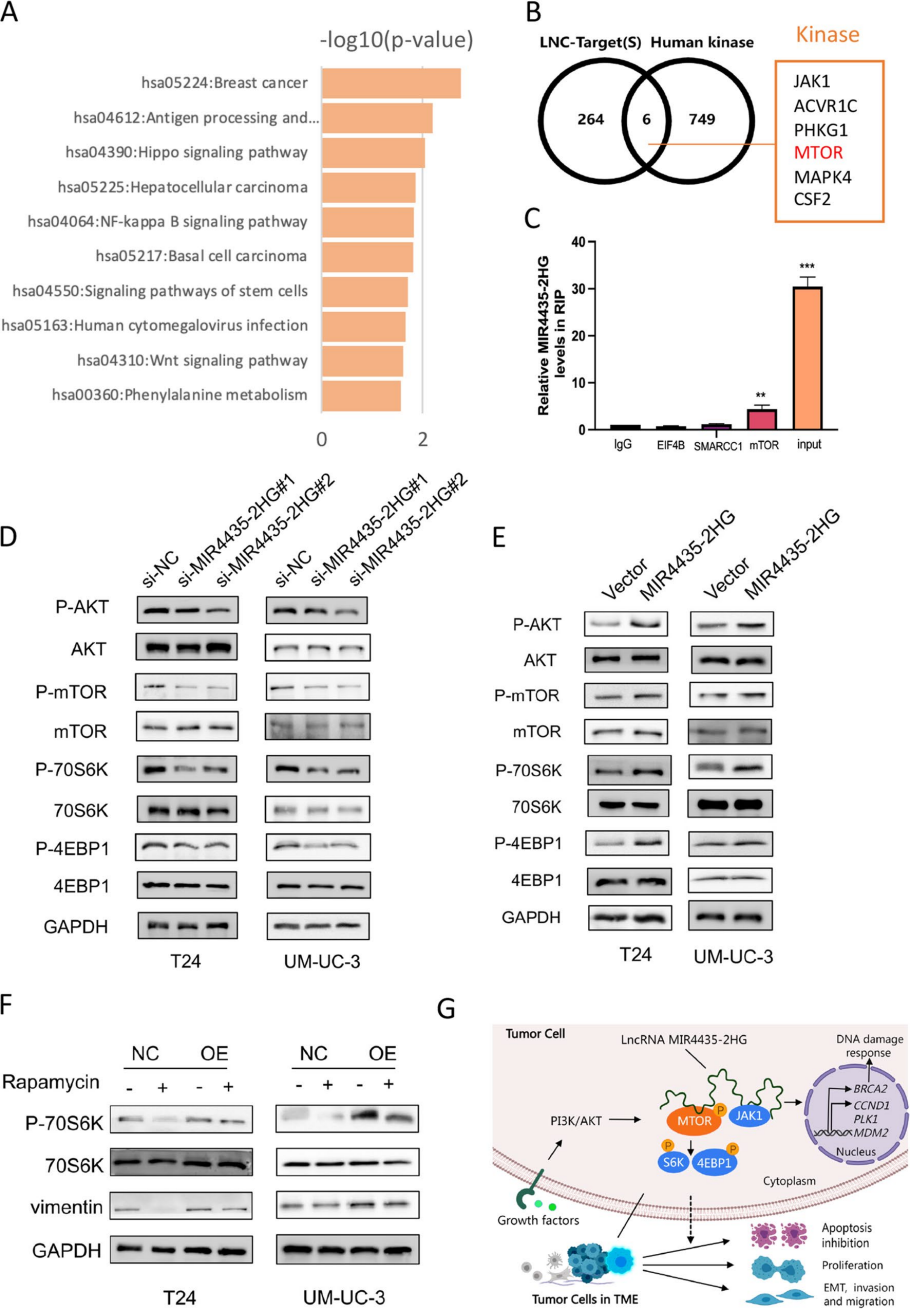
MIR4435-2HG directly interacts with mTOR to play a key role in bladder cancer. **A** Enrichment analysis of mass spectrometry data of T24 cells. **B** Kinases associated with cancer pathways among PPI proteins. **C** RIP was performed in T24 cells after which the co-precipitated RNA was subjected to qRT-PCR for MIR4435-2HG. Expression levels of MIR4435-2HG RNA are presented as fold enrichment in SMARCC1, EIF4B and mTOR relative to IgG immunoprecipitation. **D**, **E** Western blotting was performed to detected the expression of proteins in the AKT/mTOR pathway. **F** Expression of P-S6K and vimentin detected by Western blotting in T24 and UM-UC-3 cells with or without stimulation of rapamycin. **G** Mechanistic diagram of MIR4435-2HG in UCB

To identify MIR4435-2HG-interacting proteins in UCB cells, we performed RNA pull-down assays using *in vitro* transcribed biotinylated MIR4435-2HG. A mass spectrometry analysis revealed that MIR4435-2HG directly binds to more than 200 functional proteins in T24 cells (Supplementary Table S4). A subsequent protein‒protein interaction (PPI) analysis revealed that the MIR4435-2HG-interacting proteins were significantly related to protein phosphorylation, ubiquitination and methylation (Supplementary Fig. S5). An enrichment analysis of our mass spectrometry resulting genes indicated that the proteins physically interacting with MIR4435-2HG were mainly enriched in cancer-associated pathways, i.e., Hippo, NF-kappa B and Wnt signaling pathways (Fig. 7A; Supplementary Table S5). Among the PPI proteins, six kinases were found to be associated with cancer pathways, including mTOR complex 1 (mTORC1), Janus kinase 1 (JAK1), Activin receptor type 1C (ACVR1C), Phosphorylase kinase catalytic subunit gamma 1 (PHKG1) and mitogen-activated protein kinase 4 (MAPK4) (Fig. 7B). In addition, SWItch/Sucrose NonFermentable (SWI/SNF) complex proteins, i.e., SMARCA2, SMARCC1, ARID4B and ARID5A, were found to interact with MIR4435-2HG. We confirmed the physical binding of MIR4435-2HG to mTOR, EIF4B, and SMARCC1 by performing RIP assays (Fig. 7C).

Based on the important roles played by mTOR signaling in cancer progression, we next performed a Western blot analysis of mTOR pathway components. We found that the expression levels of p-Akt, p-mTOR, p-70S6K, p-4EBP1 and other proteins downstream of mTOR were significantly decreased in MIR4435-2HG knockdown UCB cells (Fig. 7D),. whereas MIR4435-2HG overexpression enhanced mTOR signaling in these cells (Fig. 7E). The protein expression of p-S6K and vimentin in T24 and UM-UC-3 cells with or without rapamycin stimulation was measured by Western blotting, showing that MIR4435-2HG regulates the effects of rapamycin (Fig. 7F).

Based on our data, we conclude that MIR4435-2HG exerts versatile functions in bladder cancer by transcriptionally regulating the expression of multiple cell-cycle regulators such as CCND1 and BRCA2. Moreover, we confirmed that MIR4435-2HG regulates mTOR signaling by directly interacting with mTOR and its related signaling molecules via phosphorylation (Fig. 7G).

## Discussion

LncRNAs are important members of the noncoding RNA family and play crucial roles in the regulation of the development and progression of human cancers [17, 18]. In the present study, we found that MIR4435-2HG, which is a novel lncRNA located at chromosome 2q13, was significantly upregulated in UCB tissues compared with normal bladder tissues. Increased MIR4435-2HG levels were found to be closely associated with a poor prognosis and short survival. Moreover, we found that MIR4435-2HG exerted versatile effects on bladder cancer,i.e., MIR4435-2HG promoted tumor cell proliferation and regulated the expression of multiple cell cycle regulators, such as CCND1 and BRCA2. Inhibiting MIR4435-2HG induced cell cycle arrest and apoptosis in bladder cancer cells. Notably, we found that MIR4435-2HG regulated mTOR and EMT signaling pathways. Our data suggest that MIR4435-2HG may be an important regulator in bladder cancer progression.

Previous studies have shown that the molecular mechanisms underlying the regulation of tumor progression by lncRNAs may involve interactions with RNA/protein targets, resulting in transcriptional (de)regulation, epigenetic alteration, sponging and modification [19, 20]. Recent studies have suggested that MIR4435-2HG may play a role in the progression of several cancer types [21–24], mainly by acting as a microRNA (miRNA) sponge. MIR4435-2HG has, for example, been found to promote hepatocellular carcinoma proliferation and metastasis by sponging miR-22-3p to liberate YWHAZ mRNA transcripts [25]. Other sponged miRNAs include miR-1224-5p [22] and miR-138-5p [26]. In addition, it has been suggested that MIR4435-2HG may regulate WNT/β-catenin signaling in tumors [27].

We found that MIR4435-2HG physically interacted with the mTORC1 complex. The mechanisms underpinning the oncogenic mode of action of MIR4435-2HG in bladder cancer cells may involve the phosphorylation of mTOR signaling molecules, in which MIR4435-2HG may act as a scaffold for the recruitment of activators. These observations may yield clues to advance the development of mTOR-based therapeutics for bladder cancer. Hartana et al*.* [28] found that MIR4435-2HG may play a role in immune cells. They reported that MIR4435-2HG may enhance immune-metabolic activities of myeloid dendritic cells (mDCs) through epigenetic regulation of a member of the mTOR signaling pathway. In addition, Yang T et al. reported that MIR4435-2HG may act as a microRNA sponge to miR-2467-3p to exert its function to enhance mTOR signaling [14]. These studies indicate that MIR4435-2HG-mediated mTOR signaling pathways may be involved in multiple molecular mechanisms.

In addition to the mTOR signaling pathway, we found that MIR4435-2HG could mediate the transcriptional activation of cell cycle signaling in bladder cancer, which may affect epigenetic modulation. We found that MIR4435-2HG physically interacted with proteins in SWI/SNF chromatin remodeling complexes including SMARCA2 and SMARCC1 (Supplementary Fig. S5). Several studies have shown that lncRNA-mediated epigenetic remodeling may play a critical role in cancer development and progression. For example, THAP7-AS1 has been found to function as an oncogenic modulator of the PI3K/AKT signaling pathway to promote the progression of gastric cancer [29]. In addition, MALAT1 bound and inactivated the pro-metastatic transcription factor TEAD, thereby preventing TEAD from associating with its coactivator YAP in breast cancer [30]. GAS5 has been reported to interact with and trigger YAP phosphorylation and degradation to inhibit the progression of colorectal cancer, and to be negatively regulated by the mA reader YTHDF3 [31]. LCAT1 has been found to regulate the function of RAC1 by sponging miR-4715-5p in lung cancer [32]. These observations suggest that the oncogenic function of MIR4435-2HG may be modulated *via* an as yet unexplored epigenetic remodeling mechanism.

Our finding that the apoptosis-related gene BRCA2 is regulated by MIR4435-2HG is particularly interesting. BRCA2 signaling is critical for DNA repair because it binds single-stranded DNA and directly interacts with the recombinase RAD51 to stimulate strand invasion, which is a vital step in homologous recombination repair. There is ample evidence showing that the BRCA2 gene is closely related to the development and prognosis of multiple tumors, such as breast cancer, ovarian cancer [33] and prostate cancer [34]. MIR4435-2HG may mediate interactions of the BRCA2-related complex in response to DNA damage. We found that BRCA2 and RAD51 were significantly upregulated in bladder cancer samples in the TCGA/BLCA RNA-Seq data, and that the expression of BRCA2 significantly correlated with that of RAD51 (Spearman R = 0.63; and p-value < 0.001). Moreover, PPI network analysis of BRCA2 and RAD51 in the STRING database (https://cn.string-db.org/) revealed that the two molecules can physically interact in cells at high confidence level (score = 0.999). These data suggest that BRCA2 and RAD51 may be up-regulated in response to DNA damage in UCB tumor tissues. Indeed, striking ER-stress in tumor may explain upregulation of BRCA2 and RAD51 expression in bladder cancer cells. However, the mechanism by which MIR4435-2HG mediates the interactions of BRCA2-related complex in tumors have yet to be explored.

We also found that MIR4435-2HG was involved in the regulation of EMT signaling pathways in UCB. A previous pan-cancer study using Gene Set Enrichment Analysis (GSEA) enrichment analysis of TCGA RNA-Seq data and Gene Expression Omnibus (GEO) microarray data suggested that MIR4435-2HG may be related to the enhancement of the EMT signaling pathway [35]. These data also support our notion based on informatics.

One of the novelties of our study is the observed correlation of MIR4435-2HG with the stroma enrichment molecular subtypes of UCB. Previous studies have indicated that the communication between tumor cells and stromal cells can be mediated by metabolic pathways [36]. Cancer cells and stromal cells may, for example, develop a complementary “symbiotic” metabolic pathway, leading to a “Reverse Warburg Effect”, affecting the consumption of lactic acid as a nutrient. Another example suggests that metabolic reprogramming of bile acids accumulated in LNs promotes LN metastasis via activation of FAO pathways induced by signaling mediated through the transcriptional coactivator YAP [37].

Based on observations on the involvement of MIR4435-2HG in MTOR and EMT signaling pathways, we speculate that MIR4435-2HG may mediate interactions between cancer cells and stromal cells by metabolic reprogramming, which may be a future direction for studies. Based on the close correlation between stromal cell enrichment and immune cell infiltration in the TME, *in vivo* models may help to more precisely characterize the dynamic interactions of tumor cells and stromal cells mediated by MIR4435-2HG.

In summary, we report that lncRNA MIR4435-2HG clinically and functionally participates in the progression of UCB. The mechanisms mediated by MIR4435-2HG may include modulation of cell cycle regulator activity and mTOR signaling in the stroma-enriched subtypes of urothelial carcinoma of the bladder. Targeting the MIR4435-2HG-associated pathways may be instrumental for the development of novel approaches for the treatment of bladder cancer.

### Supplementary Information

Below is the link to the electronic supplementary material.

Supplementary file1 (DOCX 15 KB)

Supplementary file2 (XLSX 15.5 KB)

Supplementary file3 (XLSX 9.75 KB)

Supplementary file4 (XLSX 10.2 KB)

Supplementary file5 (XLSX 78.7 KB)

Supplementary file6 (XLSX 10.4 KB)

ESM 1 (PNG 502 kb)

High resolution image (TIF 18.6 MB)

ESM 2 (PNG 558 kb)

High resolution image (TIF 61564 kb)

ESM 3 (PNG 1307 kb)

High resolution image (TIF 91769 kb)

ESM 4 (PNG 348 kb)

High resolution image (TIF 61729 kb)

ESM 5 (PNG 997 kb)

High resolution image (TIF 65307 kb)

ESM 6 (PNG 2411 kb)

High resolution image (TIF 137522 kb)

## Data Availability

Not applicable.
