# Supplementary material for: LncRNA MIR4435-2HG drives cancer progression by modulating cell cycle regulators and mTOR signaling in stroma-enriched subtypes of urothelial carcinoma of the bladder
Source: Cell Oncol (Dordr). 2023 Jun 24;46(5):1509–27. doi: 10.1007/s13402-023-00826-5 (PMC10618329; doi:10.1007/s13402-023-00826-5)
Supplement: Supplementary file 1 — Supplementary file1 (DOCX 15 KB) [file 13402_2023_826_MOESM1_ESM.docx]

**Supplementary Information for:**

**LncRNA MIR4435-2HG drives cancer progression by modulating cell cycle regulators and mTOR signaling in stroma-enriched subtypes of urothelial carcinoma of the bladder**

Lu Pei et al.

**Supplementary Methods**

**Detailed protocol of the In situ hybridization**

Slides were baked at 60°C for 2 h followed by de-waxing and hydration. Endogenous peroxidase was inactivated by incubating the sections in 3% H_2_O_2_ for 30 min. Added a total of 1 ml pepsin diluted with 3% citric acid, and digested at 37°C for 30 min, and then washed with PBS 3 times in 5 min. Subsequently, added a total of 20 μl pre-hybridization solution, and incubated at 37°C for 4 h, and then hybridized with 20 μl MIR4435-2HG hybridization solution with a concentration of 2 μg/ml, and incubated at 37°C overnight. Then, the sections were washed by 2×SSC solution at 37°C twice in 5 min and then washed by 0.5×SSC solution at 37°C for 15 min, and also washed by 0.2×SSC solution at 37°C for 15 min. Blocking solution was added dropwise and incubated at 37°C for 30 min, then biotinylated mouse anti digoxin was added, incubated at 37°C for 60 min, and washed with PBS 4 times in 5 min. SABC was added dropwise and incubated at 37°C for 20 min, washed three times with PBS for 5 min, and then biotinylated peroxidase was added, incubated at 37°C for 20 min, and washed three times with PBS in 5 min. Stained with 50 μL DAB solution for 10 min, washed with ddH_2_O, and then restained with hematoxylin.

**Supplementary Tables and Supplementary Figures**

**Supplementary Tables:**

Supplementary Table S1. Clinical Information of the studied subjects.

Supplementary Table S2. The sequences of the primers used in the study.

Supplementary Table S3. Antibodies used in the study.

Supplementary Table S4. MS analysis of MIR4435-2HG-sense pull-down proteins exclude MIR4435-2HG-antisense pull-down proteins in T24 cells.

Supplementary Table S5. Enrichment analysis of the binding proteins of MIR4435-2HG identified in the MS assay.

**Supplementary Figures:**

**Supplementary Figure S1**. Correlation analysis of MIR4435-2HG gene expression and the molecular subtypes of UCB. **A**. The expression of MIR4425-2HG was negatively correlated with the Luminal Papillary subtype markers PPARG and GATA3. **B**. The expression of MIR4435-2HG was positively correlated with the stroma markers POSTN and FN1. **C**. The expression of MIR4435-2HG was positively correlated with the immune cell markers PDCD1 and CXCL10.

**Supplementary Figure S2**. Correlation analysis of the expression of MIR4435-2HG and EMT genes. **A**. The expression of MIR4435-2HG was positively correlated with the EMT-associated lncRNA LINC00152. **B**. The expression of MIR4435-2HG was positively correlated with the EMT-associated marker VIM. **C**. K-M plot for survival curves of the expression levels of lncRNA LINC00152. **D**. K-M plot for survival curves of the e expression levels of EMT gene VIM.

**Supplementary Figure S3**. Construction of the model based on MIR4435-2HG-correlated genes for the prediction of the UCB survival in the TCGA dataset. **A**. Dimension reduction of the MIR4435-2HG-correlated genes using LASSO approach. **B**. The heatmap of the RNA expression for the MIR4435-2HG-correlated genes used in the predictive model. **C**. The gene expression of predictors in the high- and low-riskscore groups. **D**. Scatter plots for the correlation of the gene expression for the predictors and the expression levels of MIR4435-2HG. Note, only ECM1, EFEMP1, and FER1L4 are shown.

**Supplementary Figure S4**. RNA-Seq analysis of the dysregulated genes in the UC cells with knockdown of MIR4435-2HG.

**Supplementary Figure S5**. Illustration of the PPI-network of the proteins interacting with MIR4435-2HG detected by RNA-pulldown and subsequent MS assay. Note, only proteins with high confidence with interaction score >0.7 are shown.
