# Supplementary figures and images for: LncRNA MIR4435-2HG drives cancer progression by modulating cell cycle regulators and mTOR signaling in stroma-enriched subtypes of urothelial carcinoma of the bladder
Source: Cell Oncol (Dordr). 2023 Jun 24;46(5):1509–27. doi: 10.1007/s13402-023-00826-5 (PMC10618329; doi:10.1007/s13402-023-00826-5)

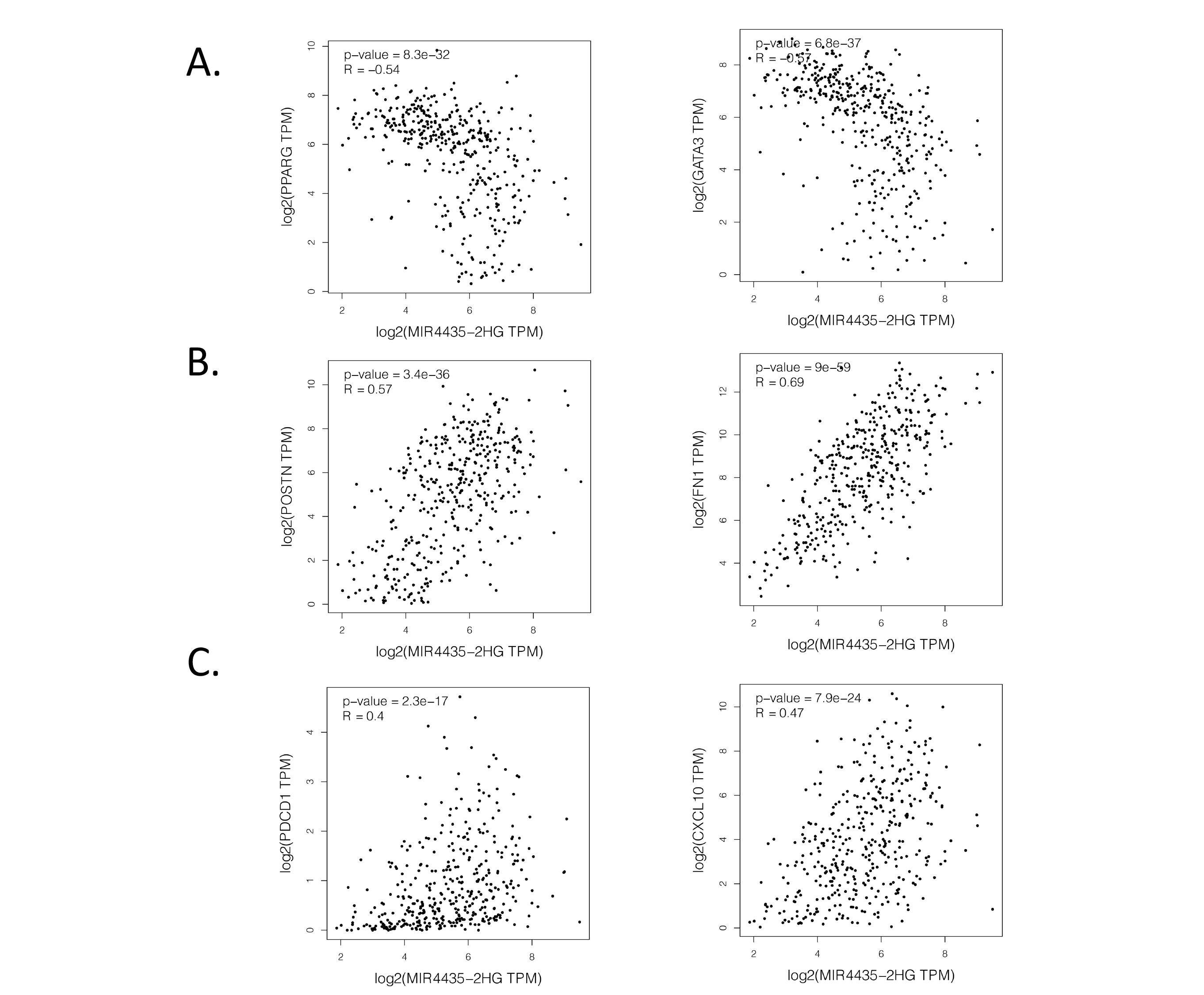

Supplement: Supplementary file 7 — (PNG 502 kb) [file 13402_2023_826_Fig8_ESM.png]

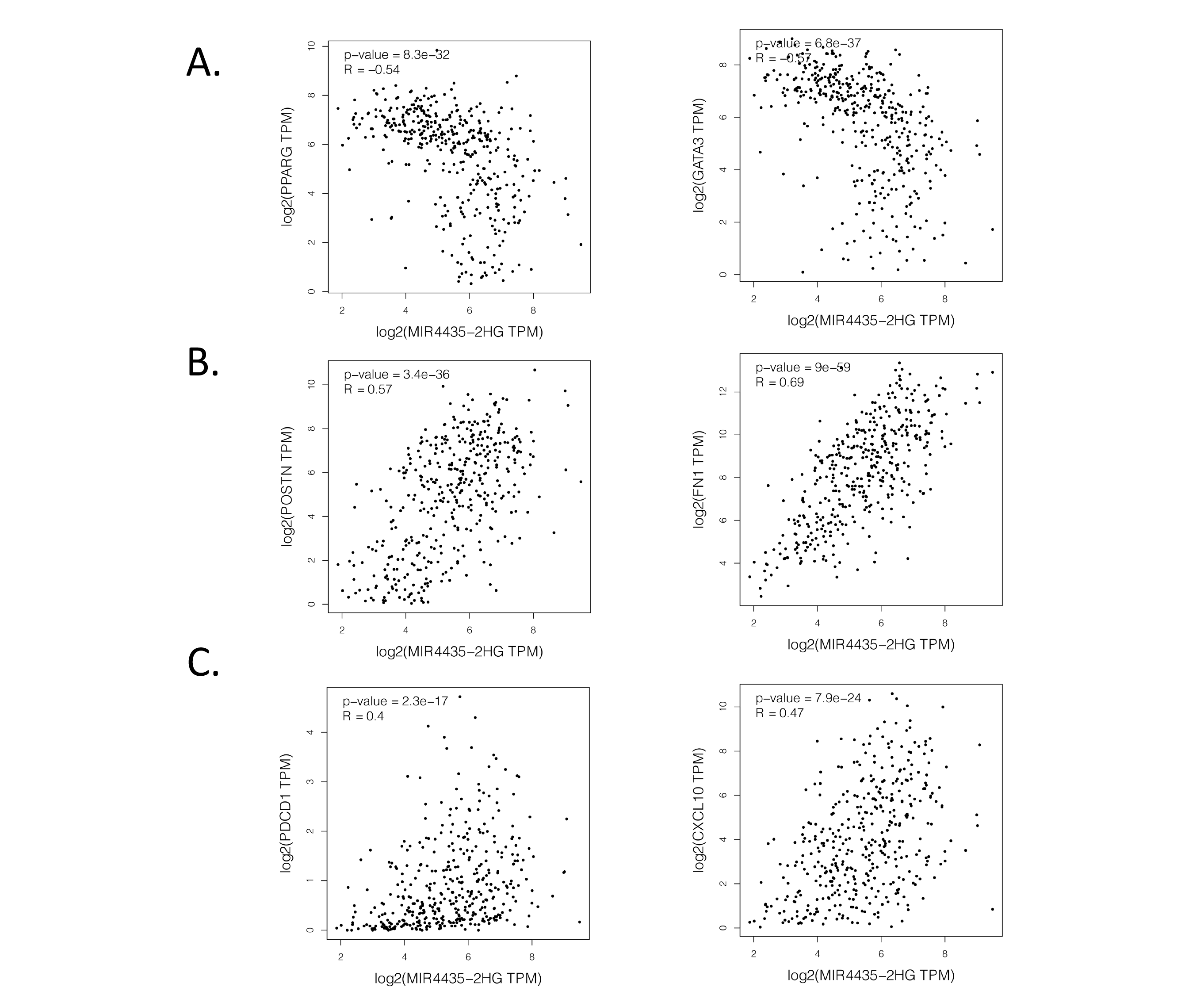

Supplement: Supplementary file 8 — High resolution image (TIF 18.6 MB) [file 13402_2023_826_MOESM7_ESM.tif]

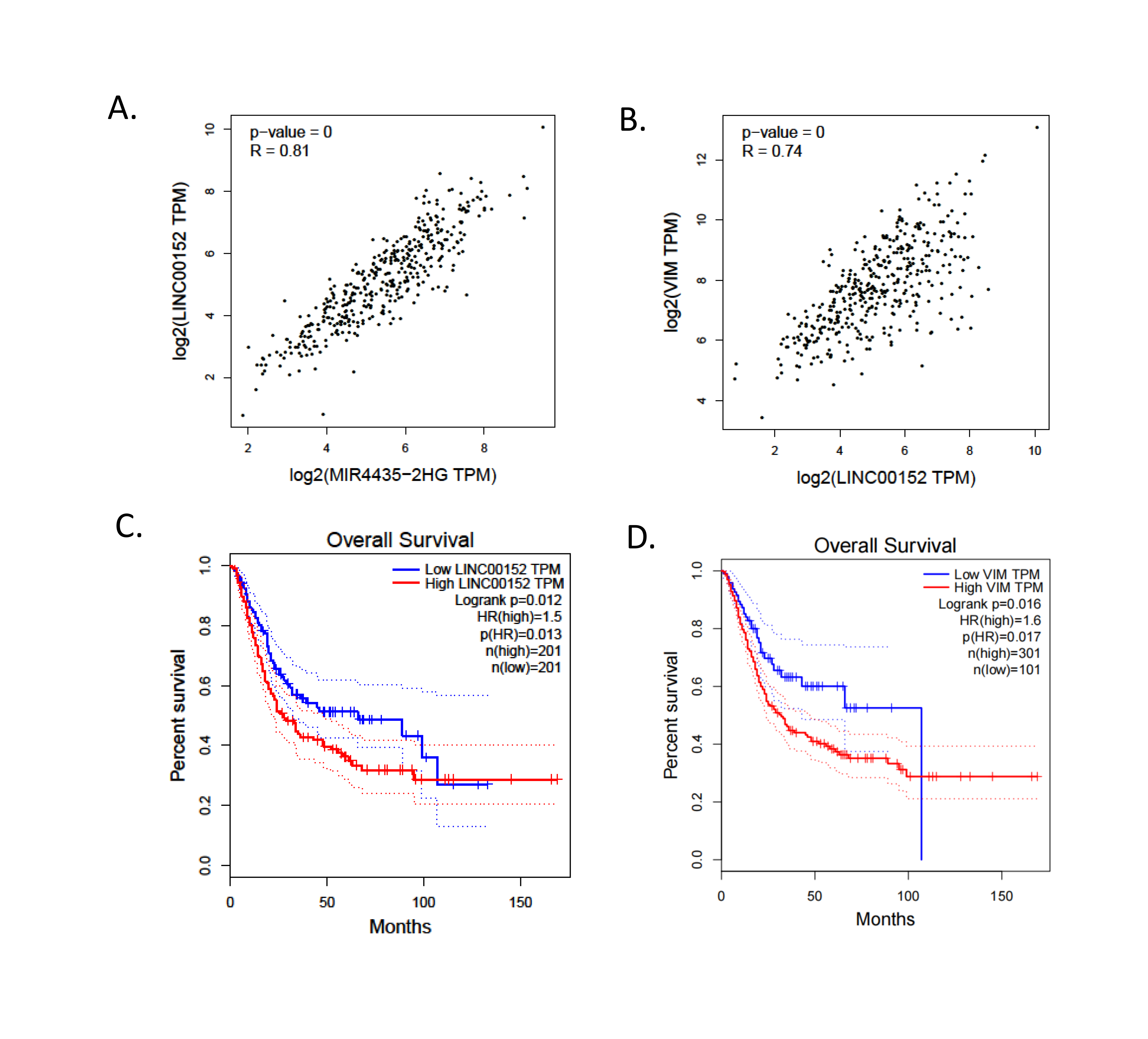

Supplement: Supplementary file 9 — (PNG 558 kb) [file 13402_2023_826_Fig9_ESM.png]

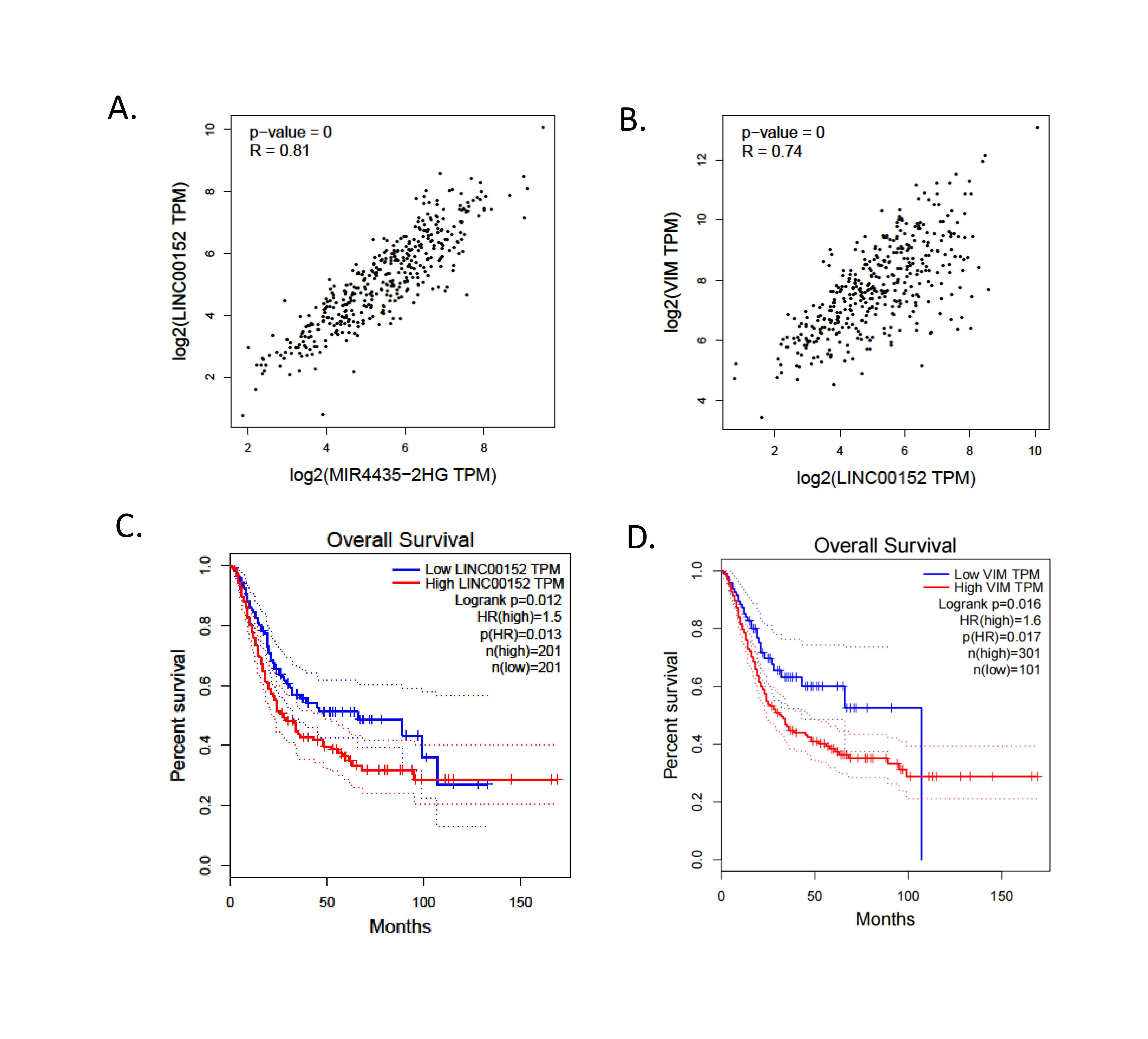

Supplement: Supplementary file 10 — High resolution image (TIF 61564 kb) [file 13402_2023_826_MOESM8_ESM.tif]

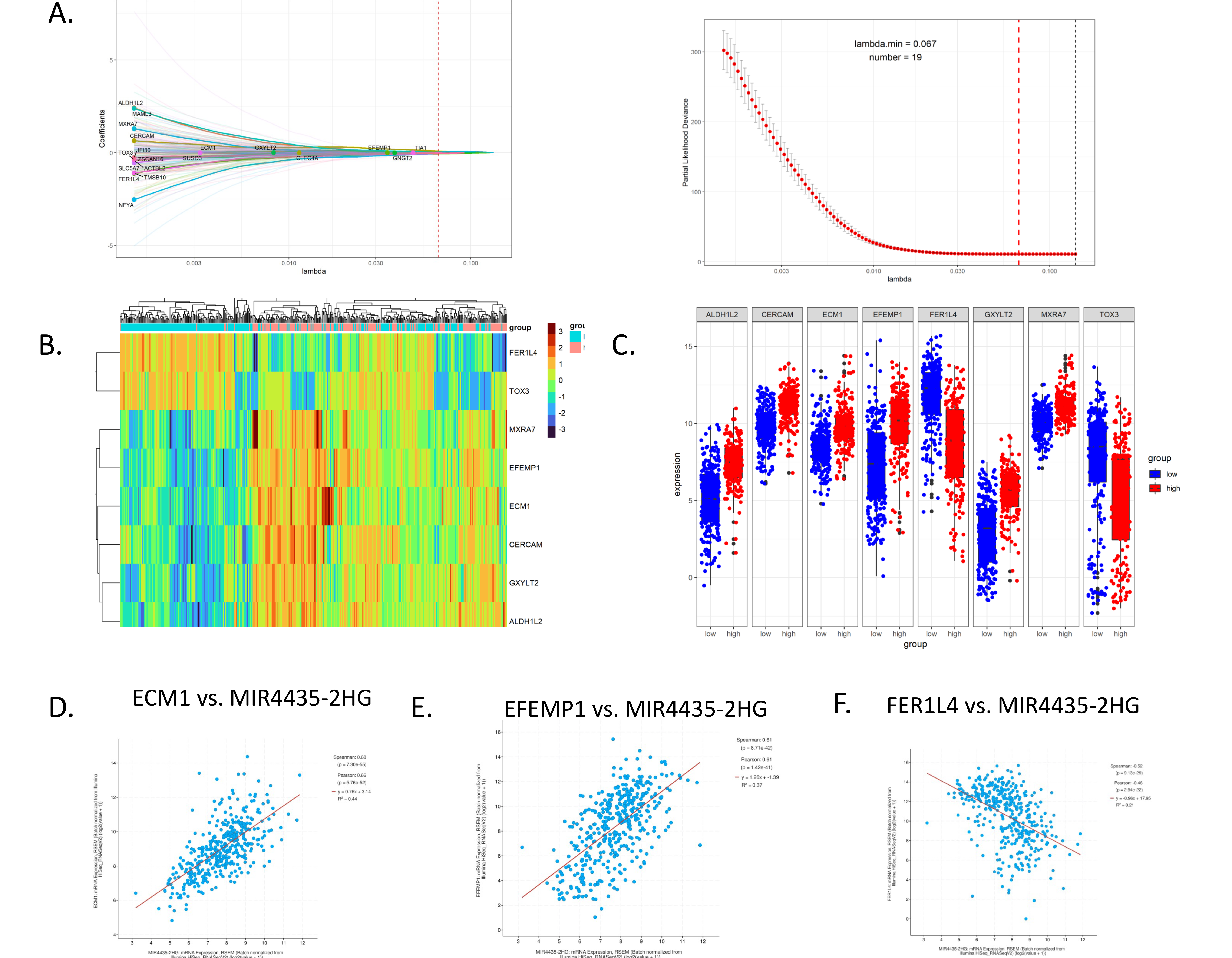

Supplement: Supplementary file 11 — (PNG 1307 kb) [file 13402_2023_826_Fig10_ESM.png]

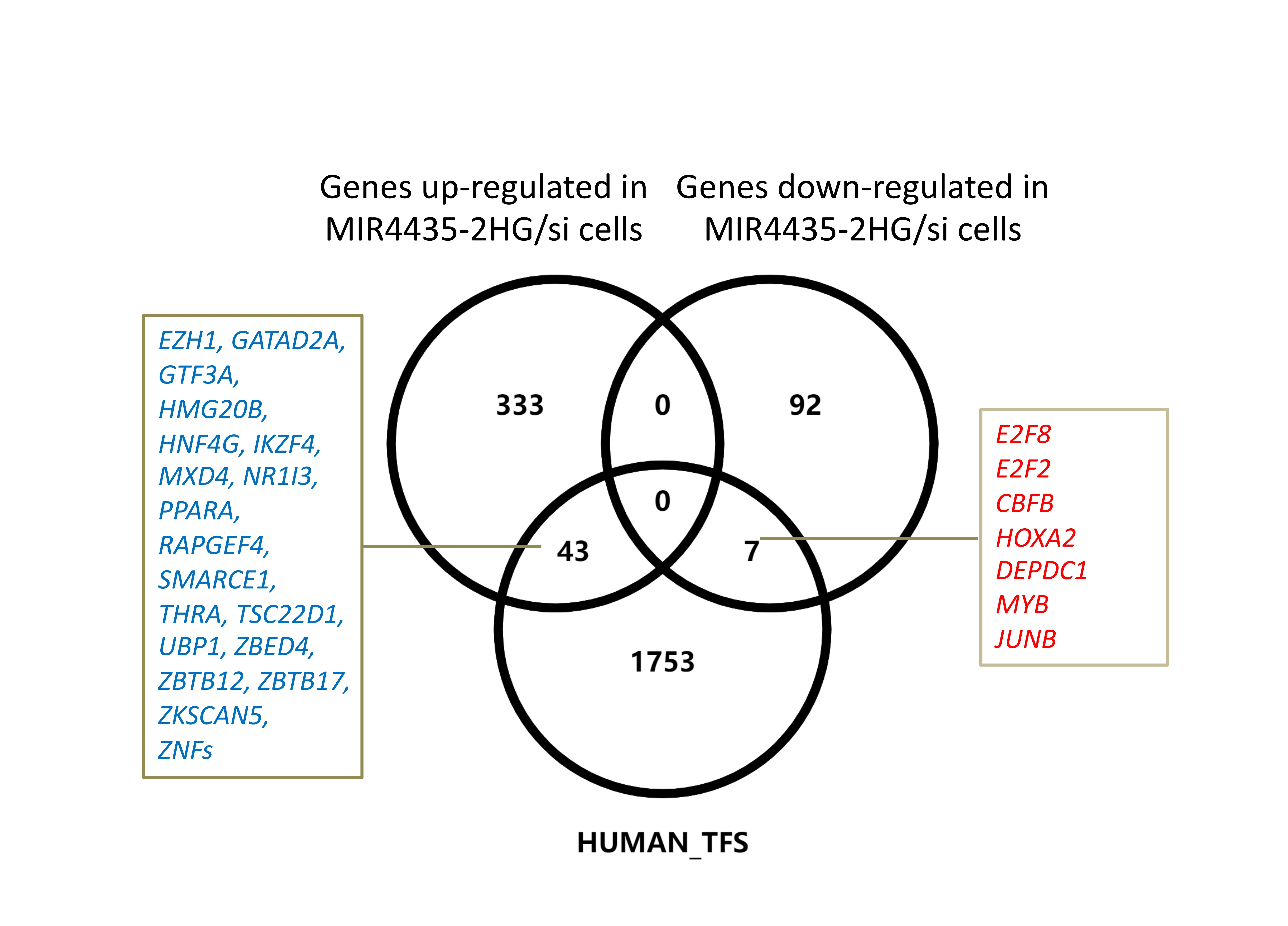

Supplement: Supplementary file 13 — (PNG 348 kb) [file 13402_2023_826_Fig11_ESM.png]

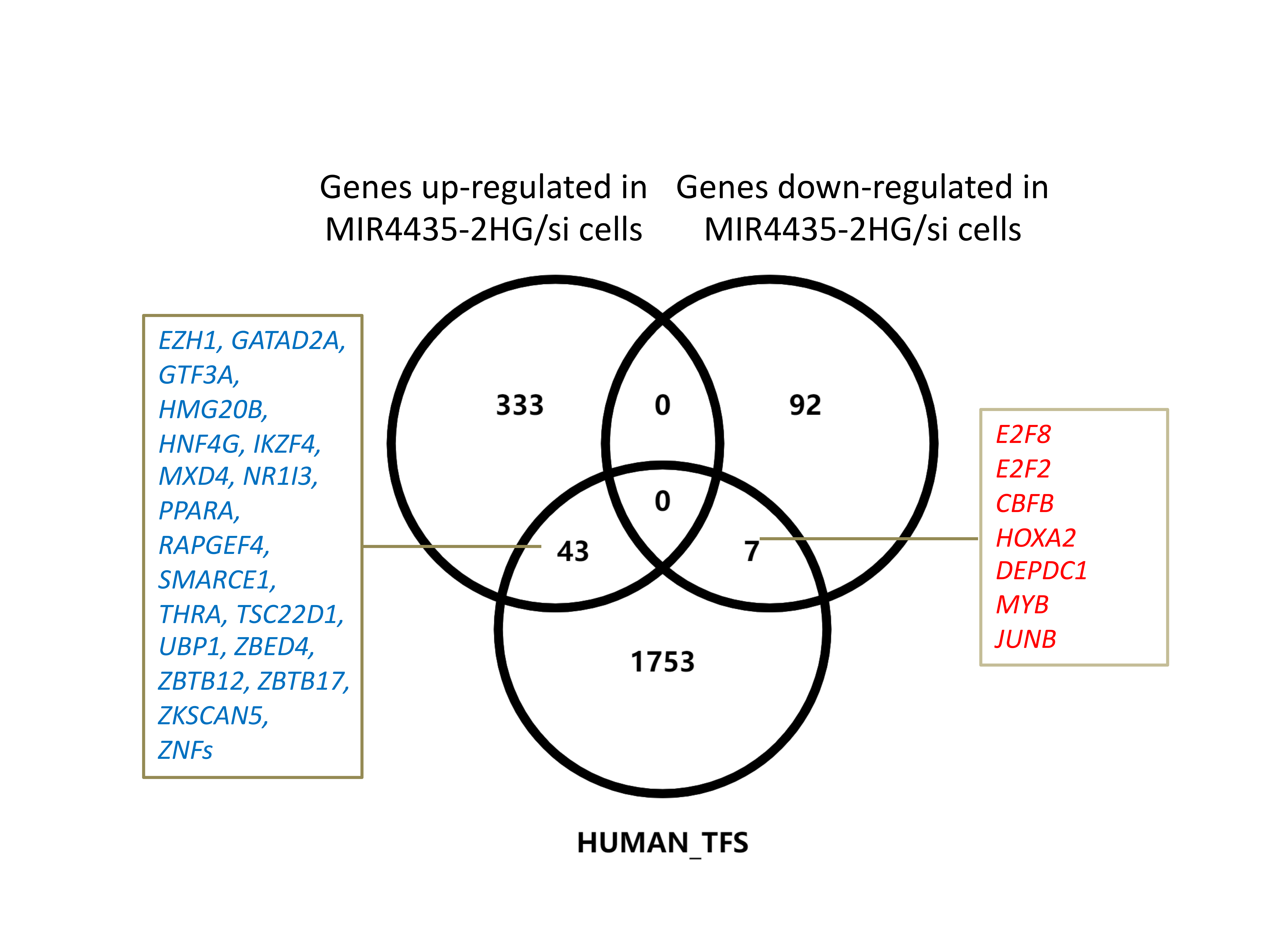

Supplement: Supplementary file 14 — High resolution image (TIF 61729 kb) [file 13402_2023_826_MOESM10_ESM.tif]

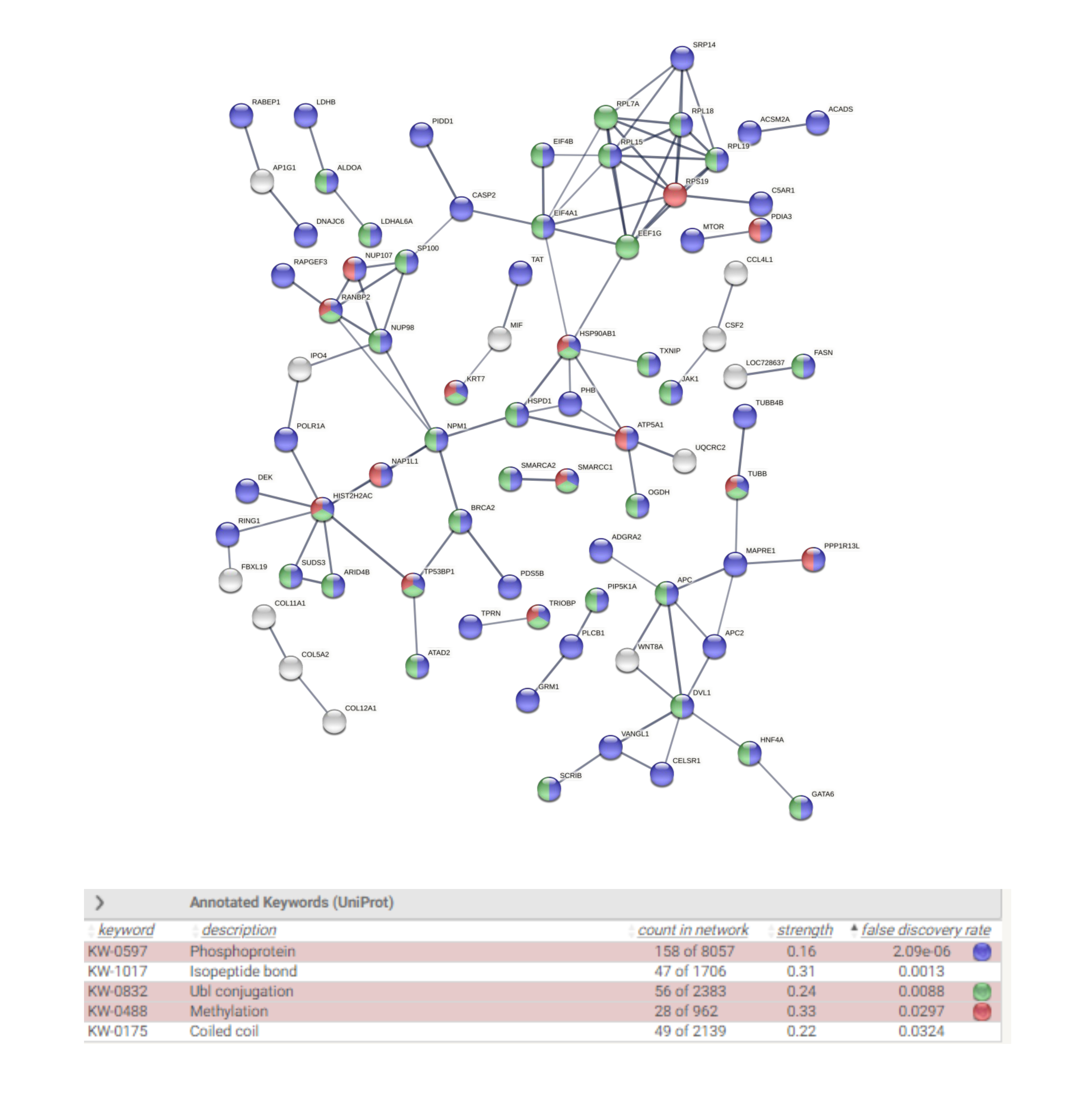

Supplement: Supplementary file 15 — (PNG 997 kb) [file 13402_2023_826_Fig12_ESM.png]

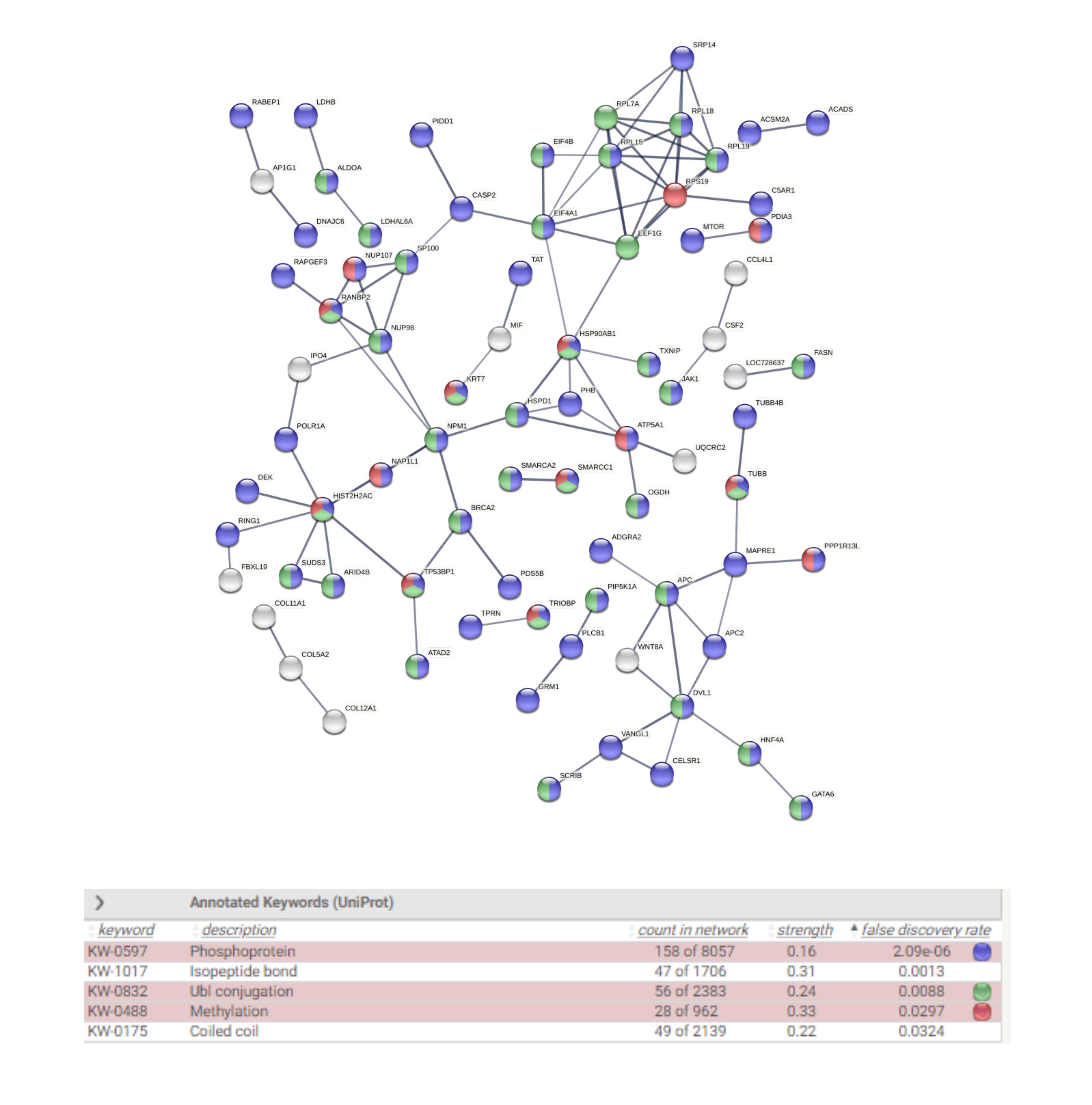

Supplement: Supplementary file 16 — High resolution image (TIF 65307 kb) [file 13402_2023_826_MOESM11_ESM.tif]

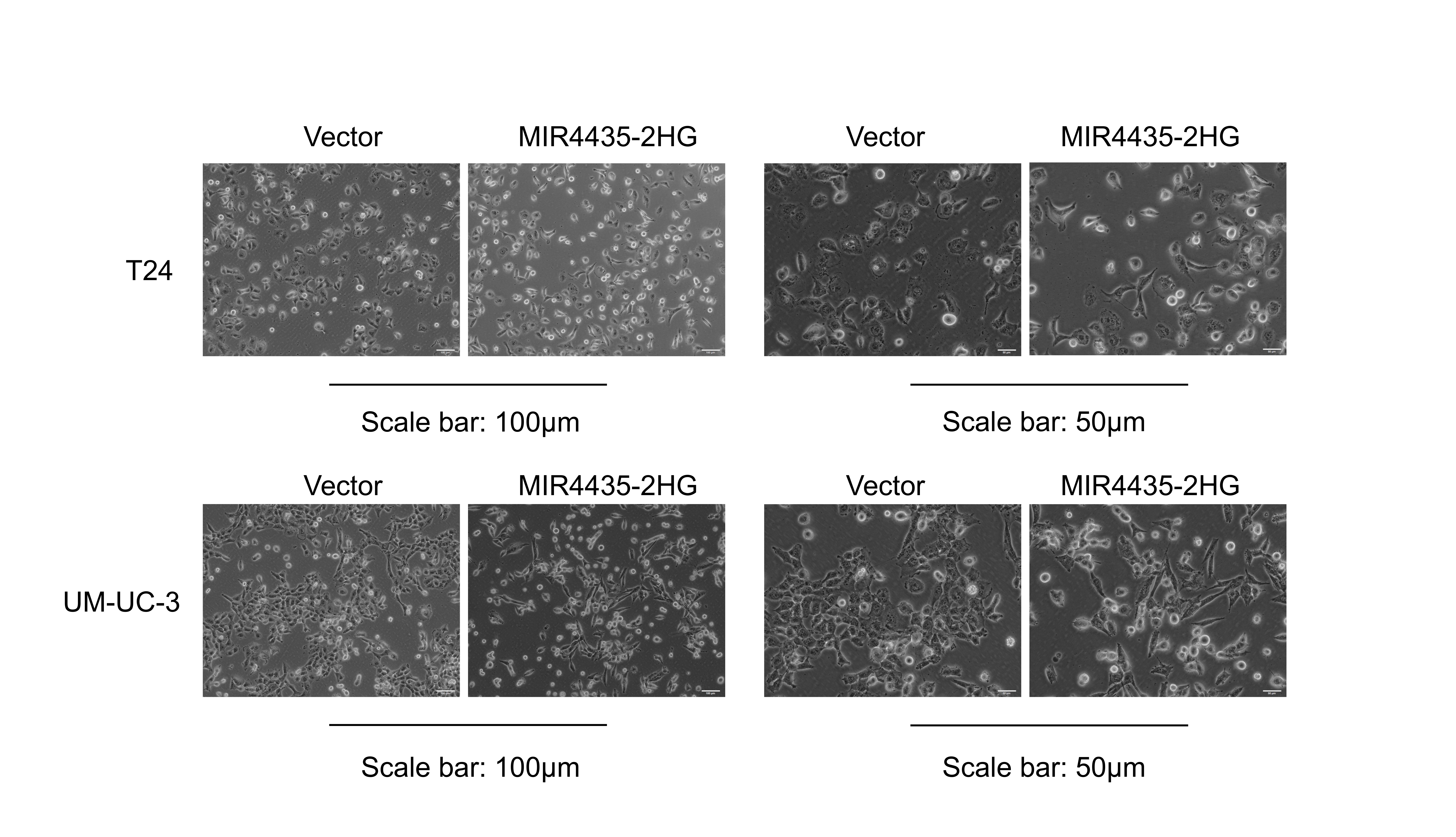

Supplement: Supplementary file 17 — (PNG 2411 kb) [file 13402_2023_826_Fig13_ESM.png]
